# Supplementary material for: Inhibition of ULK1/2 and KRASG12C controls tumor growth in preclinical models of lung cancer
Source: eLife. 2024 Aug 30;13:RP96992. doi: 10.7554/eLife.96992 (PMC11364435; doi:10.7554/eLife.96992)

**Supplemental Figure 2 G:**

pAKT


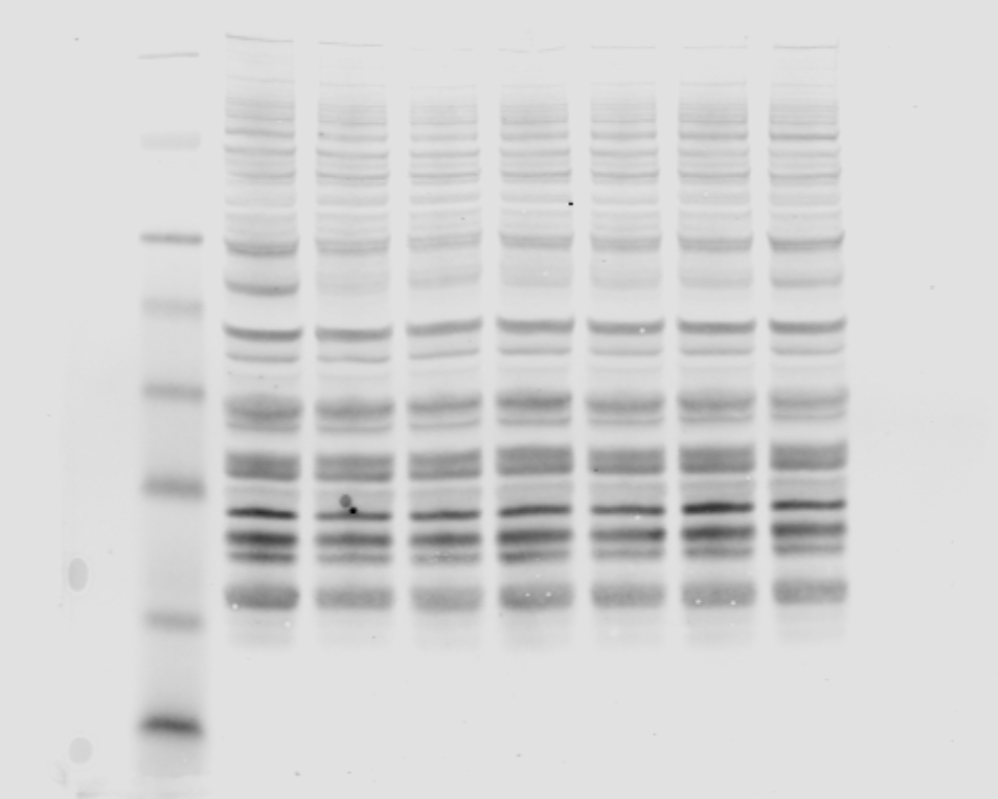


tAKT


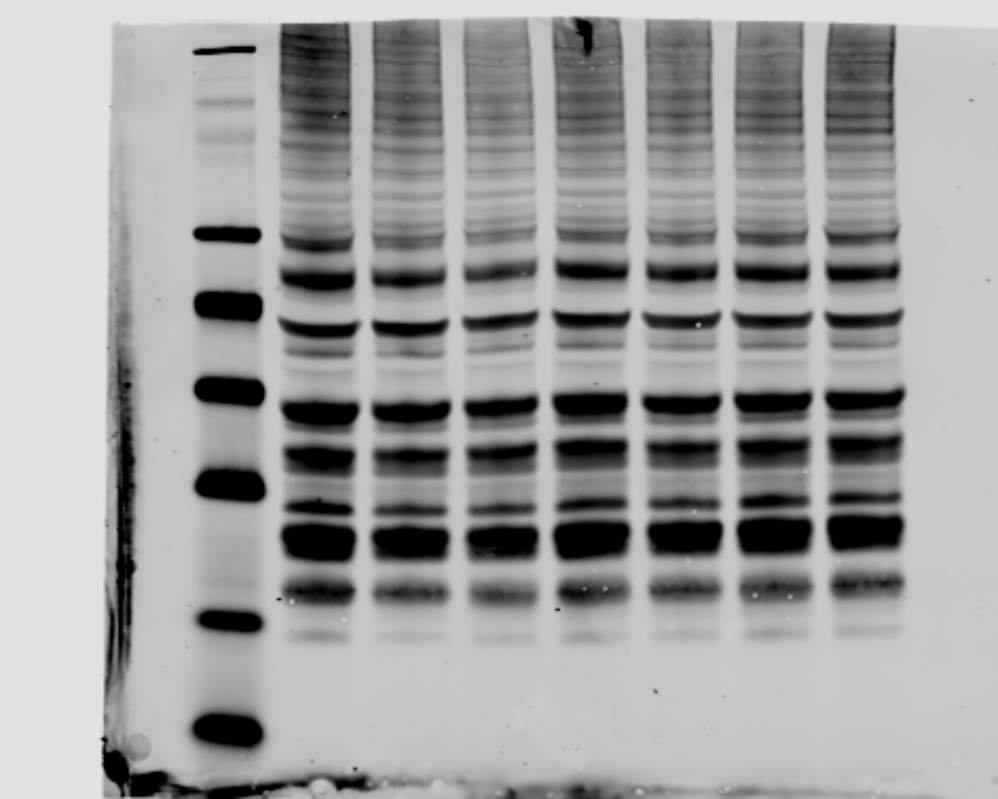


pERK


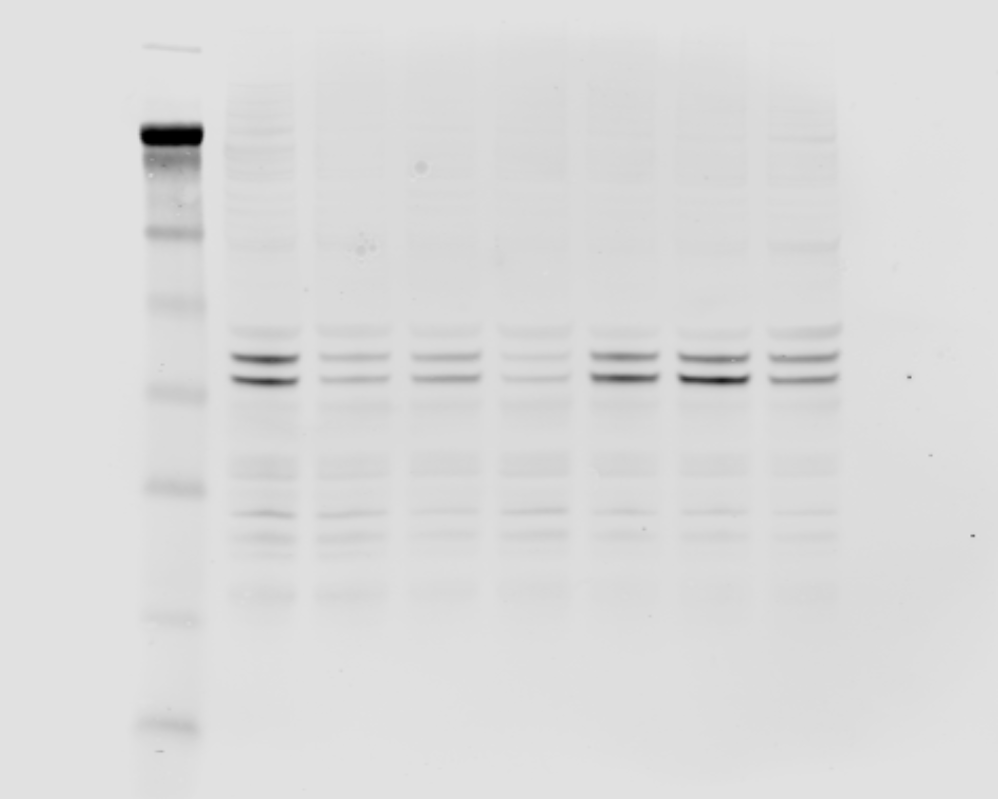


tERK


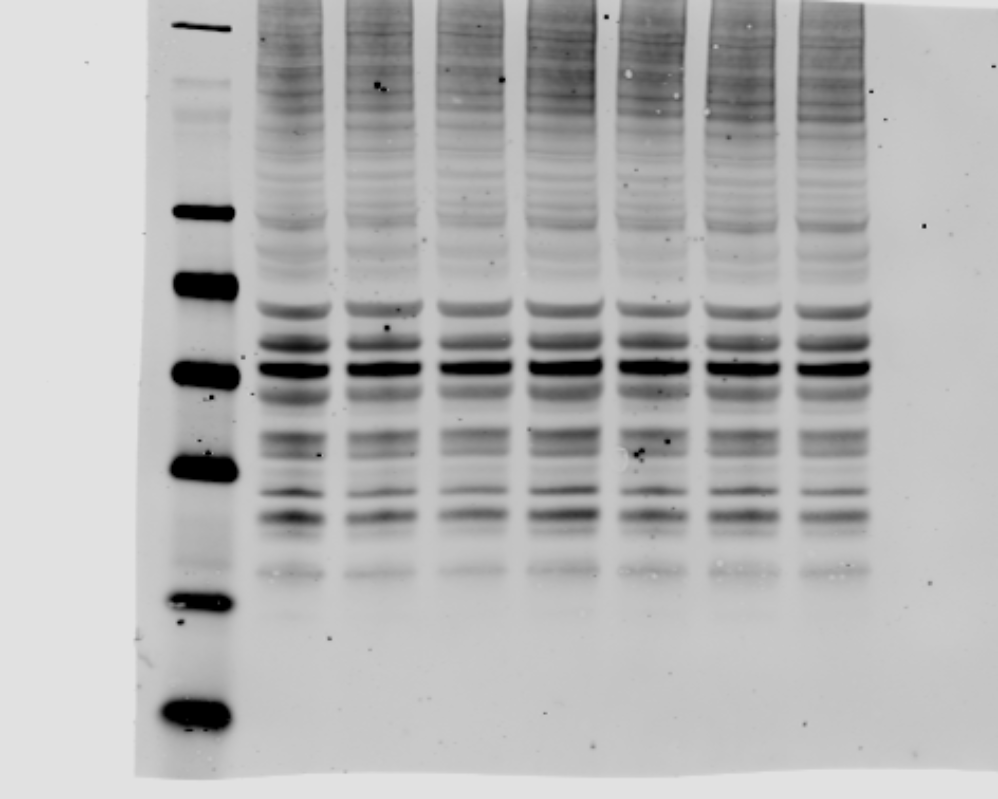


LC3A/B


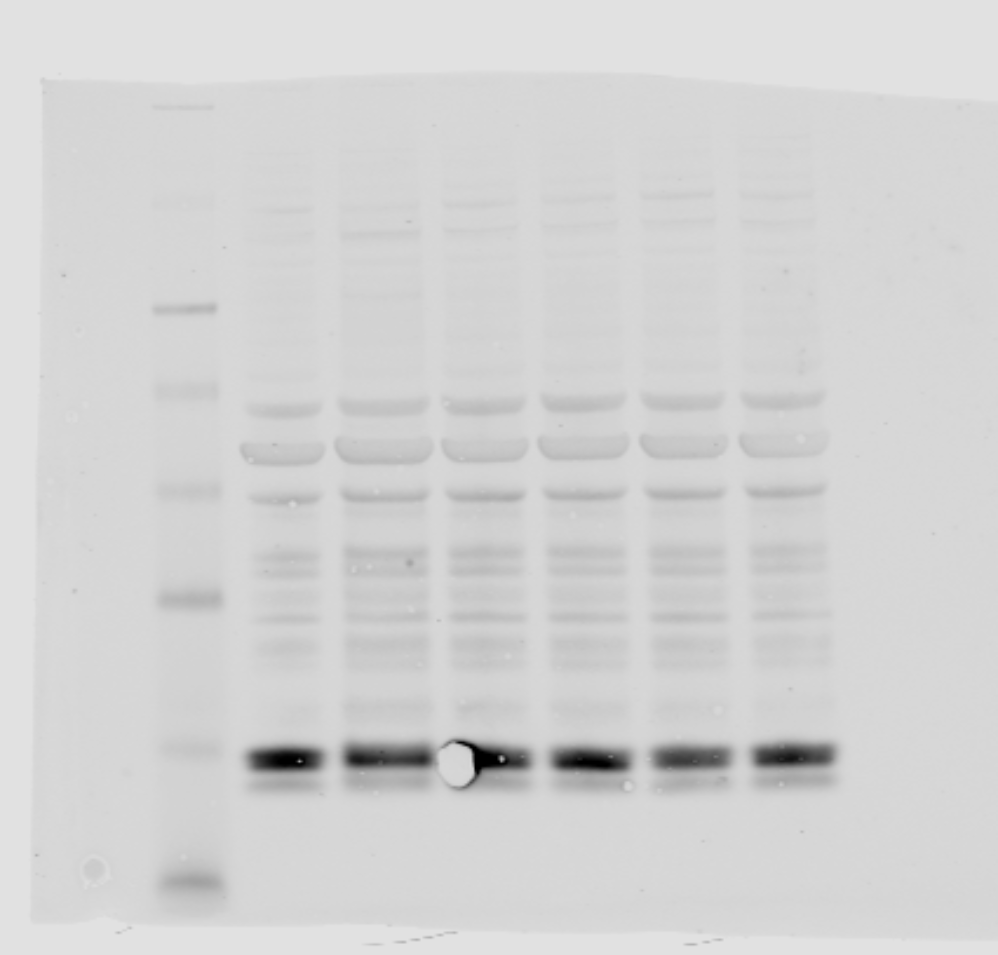


RAS


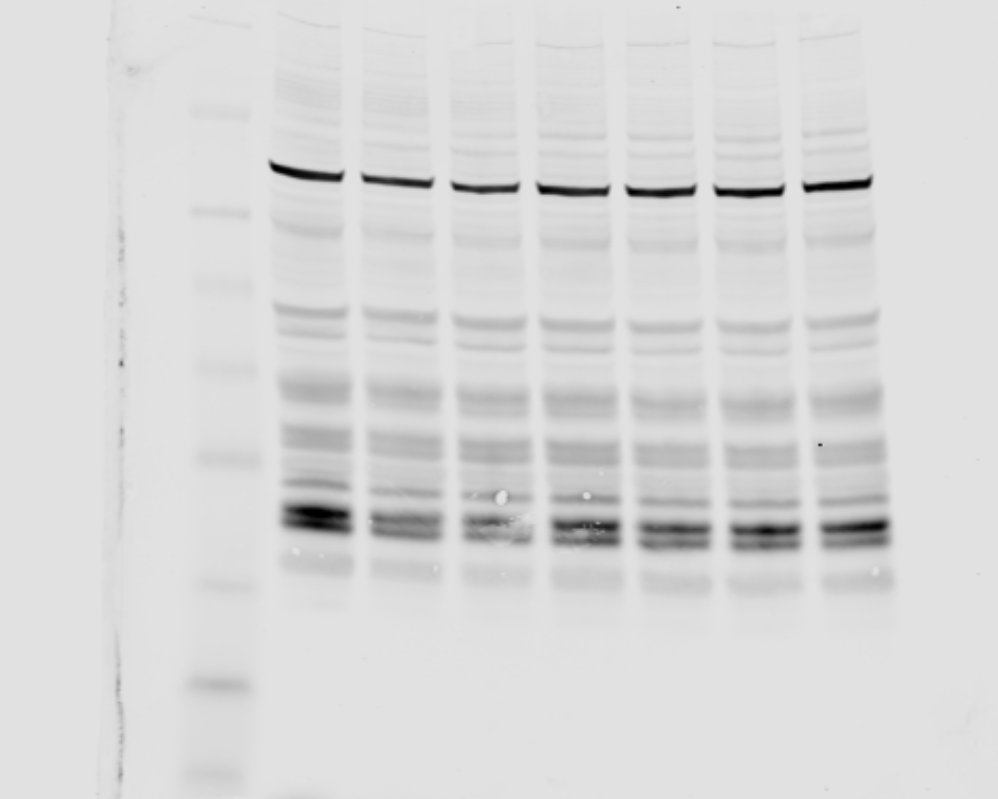


b-actin


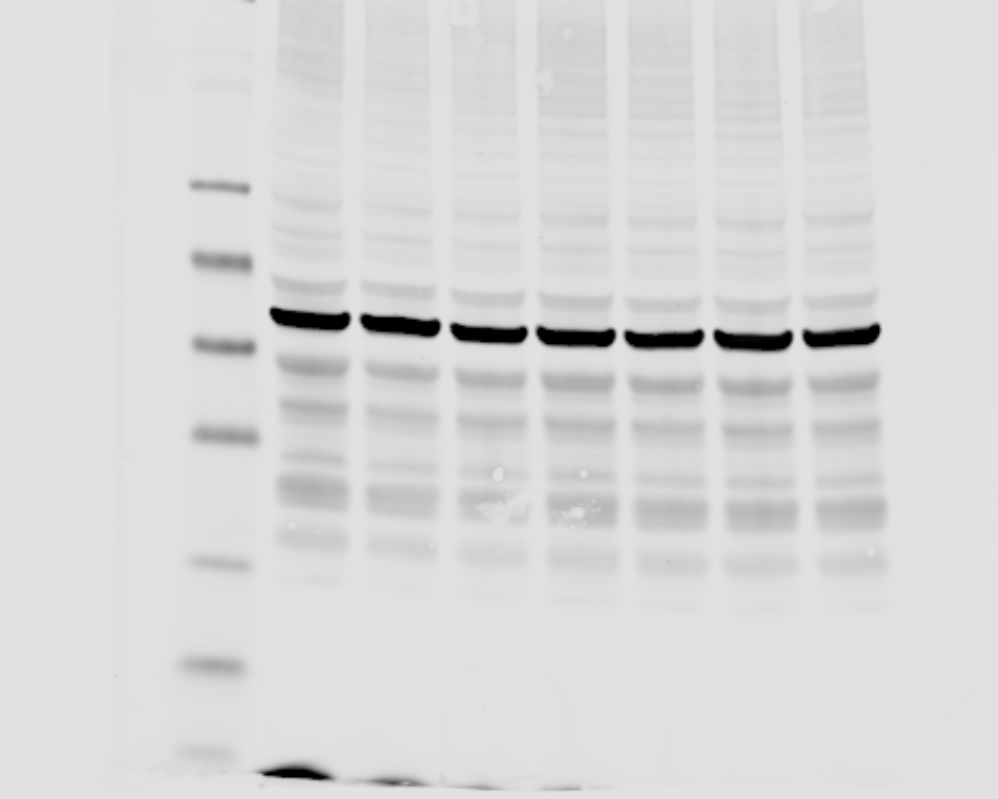


Supplemental Figure 2 H:

pAKT


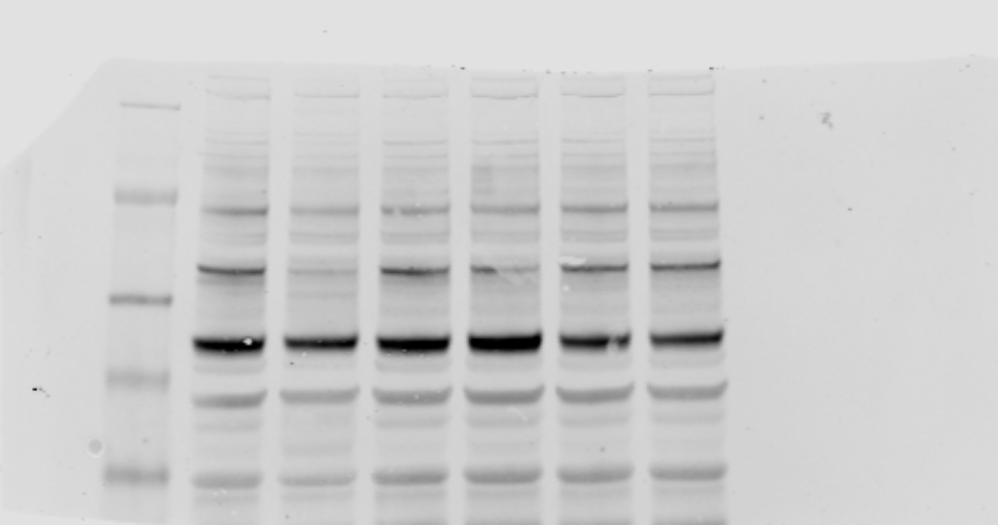


tAKT


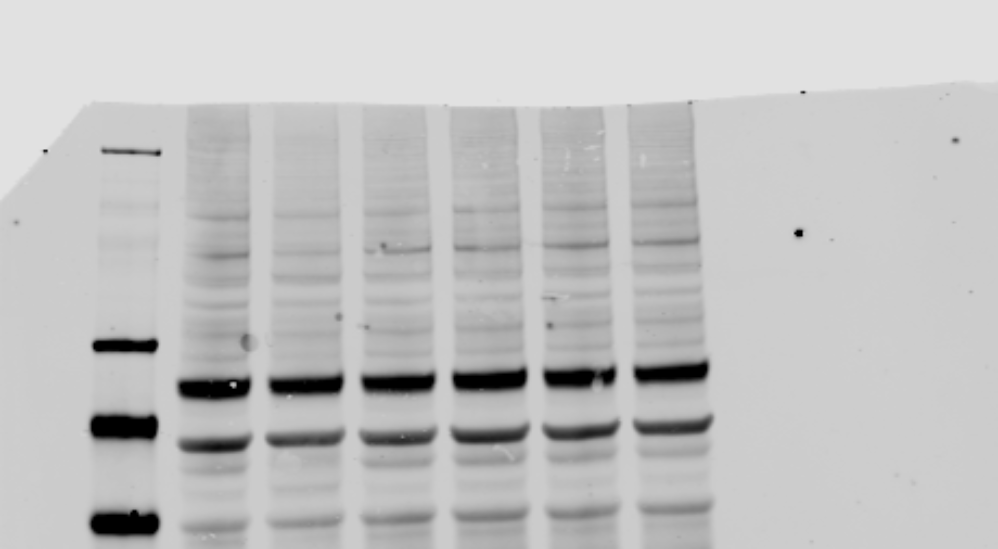


pERK


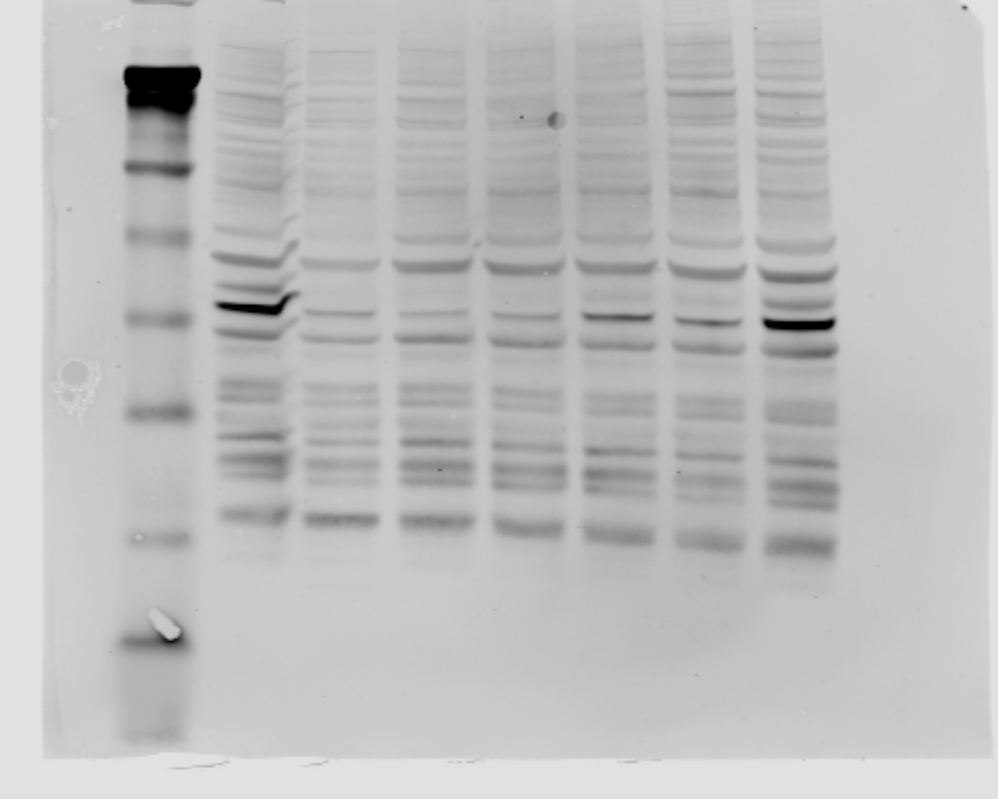


tERK


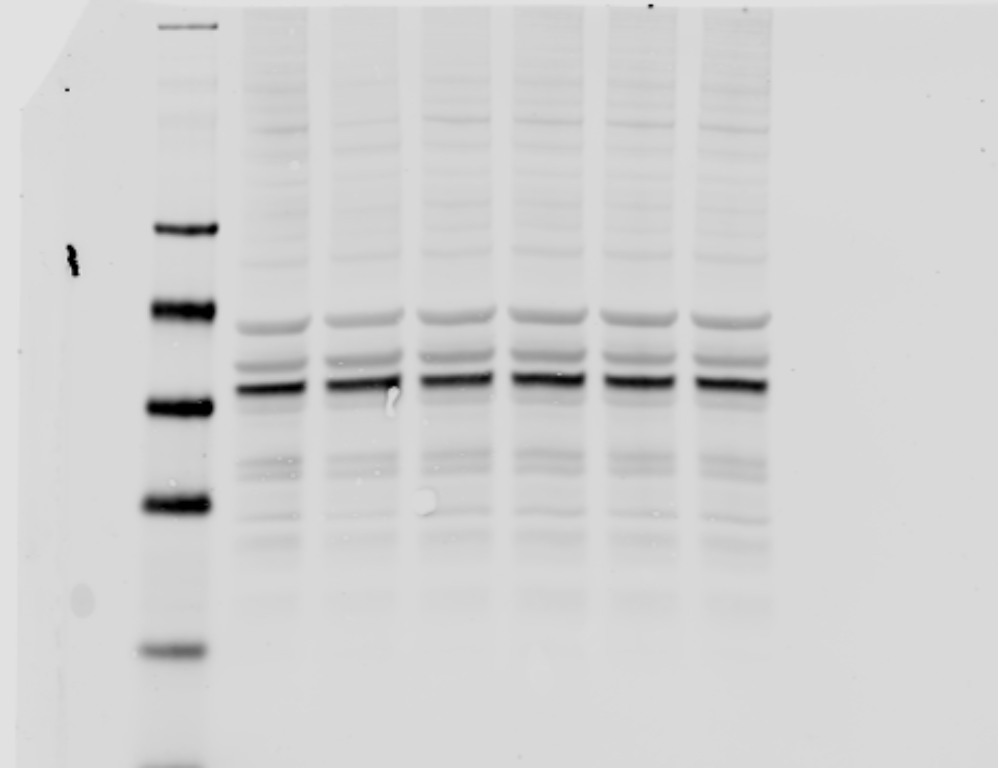


LC3A/B


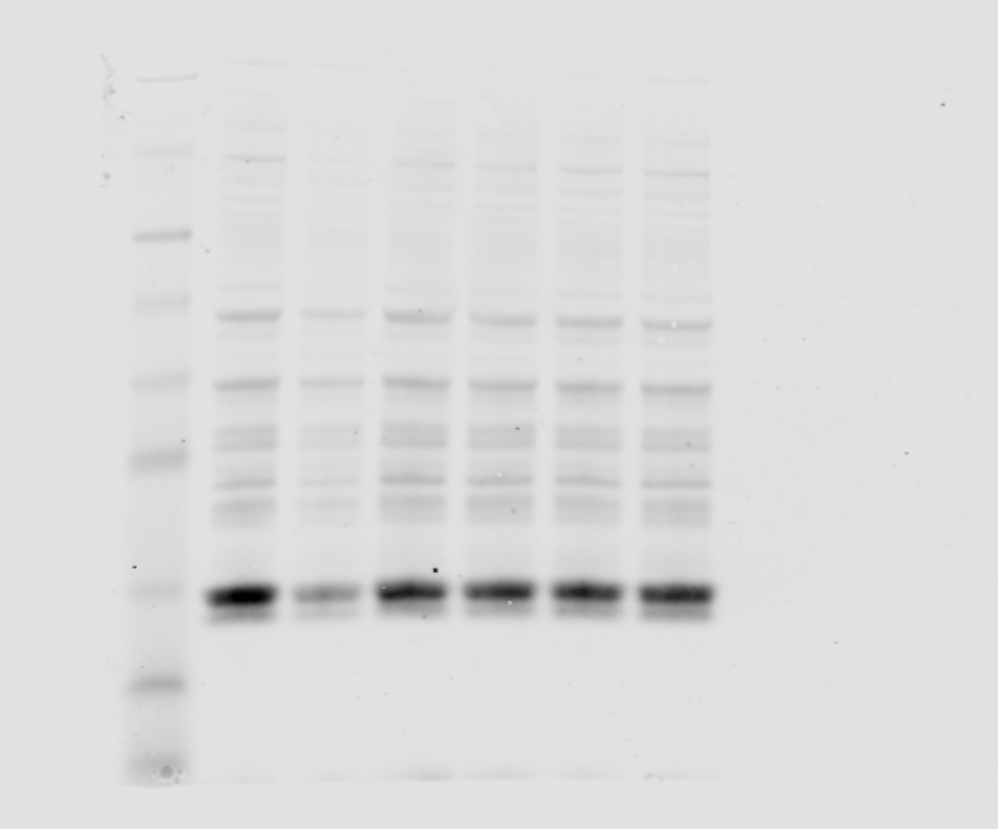


RAS


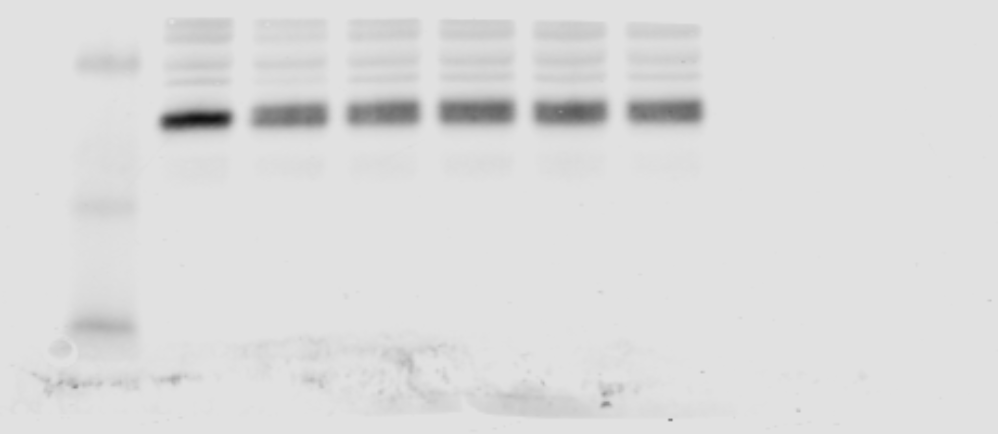


b-actin


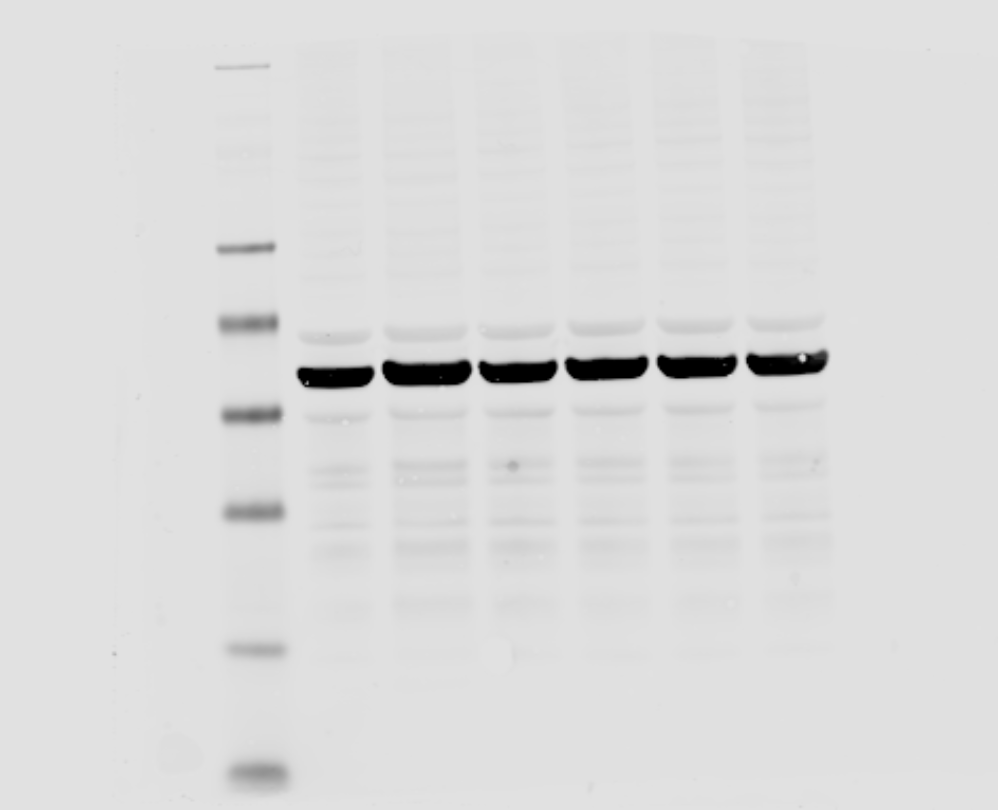


Supplemental Figure I:

pAKT


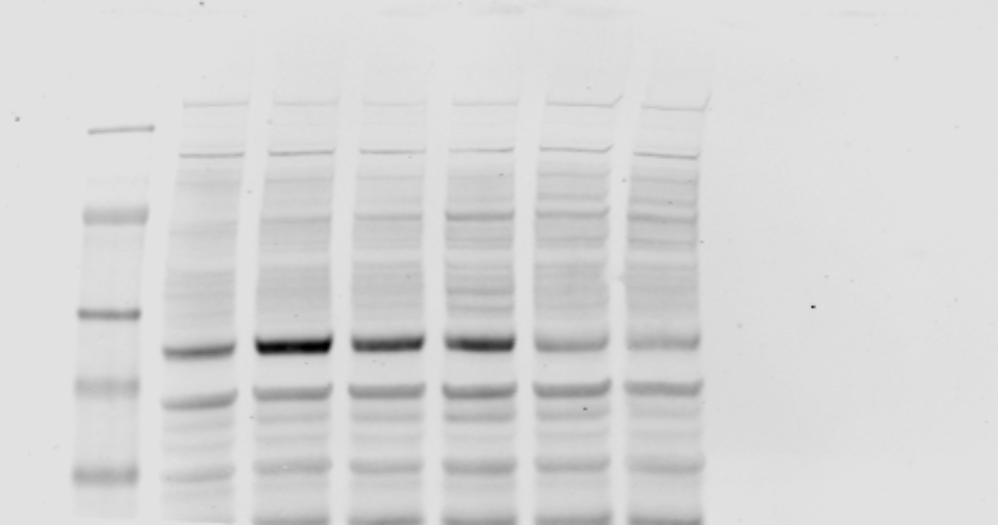


tAKT


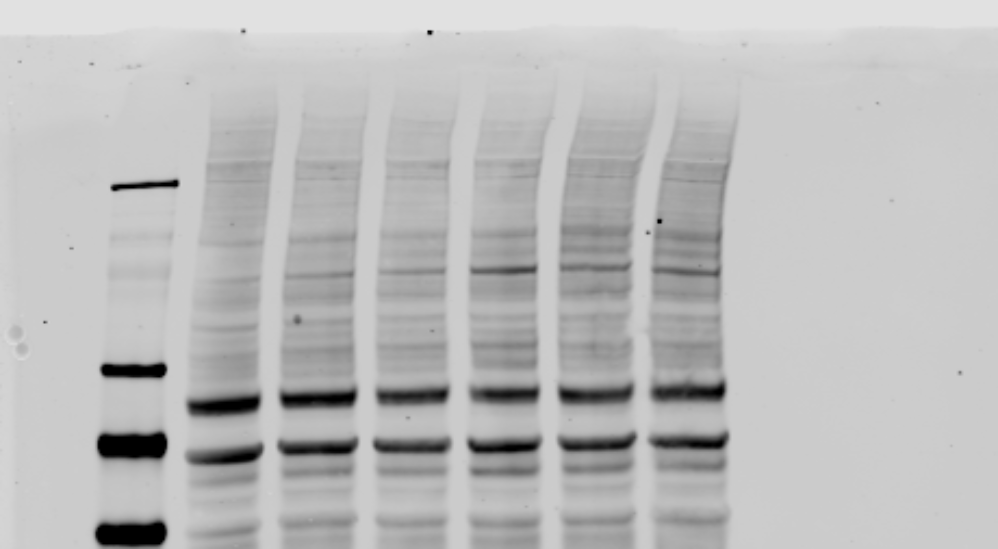


pERK


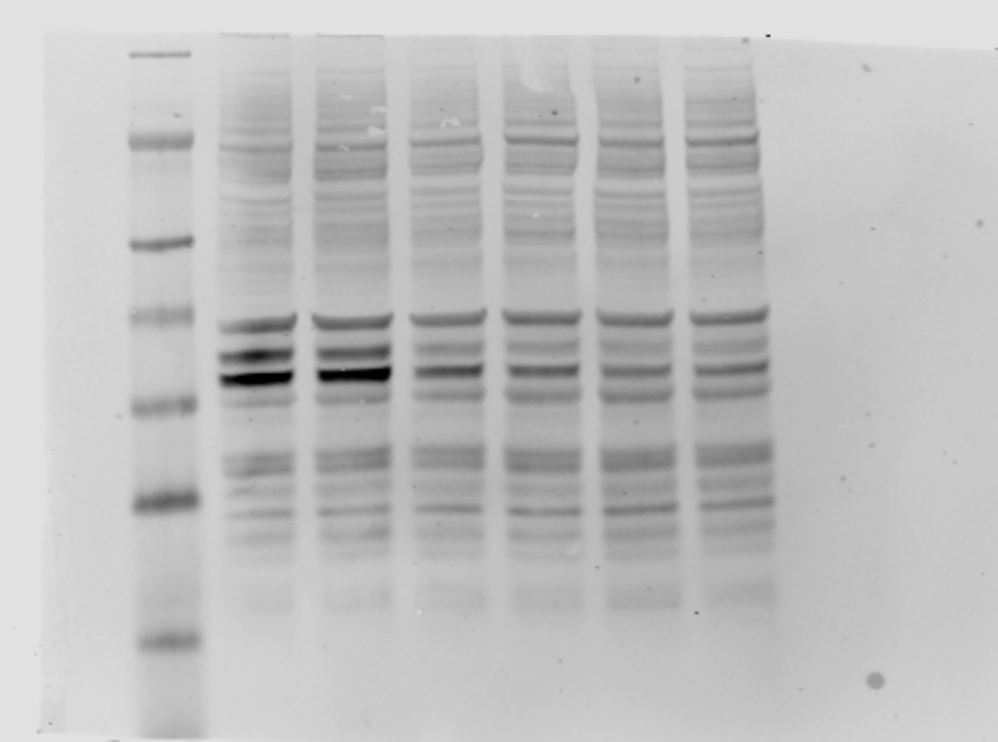


tERK


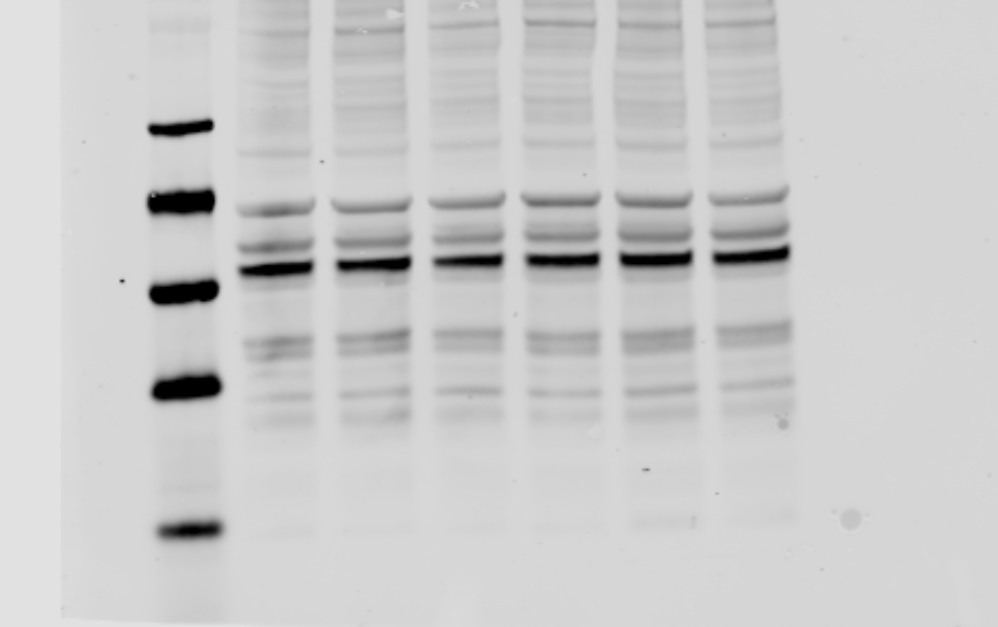


LC3A/B


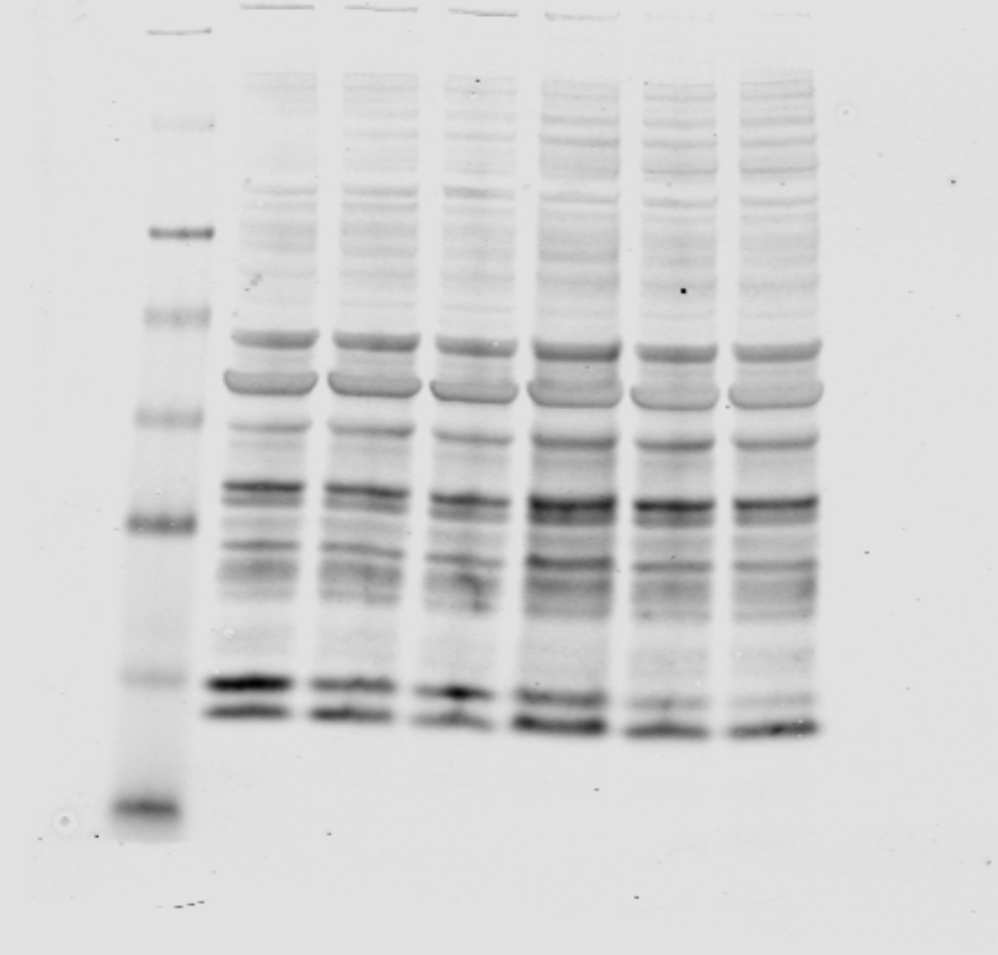


RAS


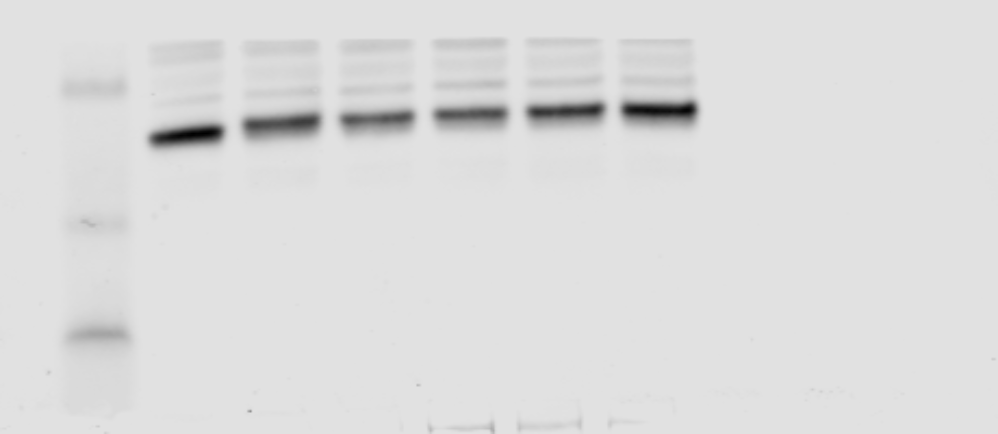


b-actin


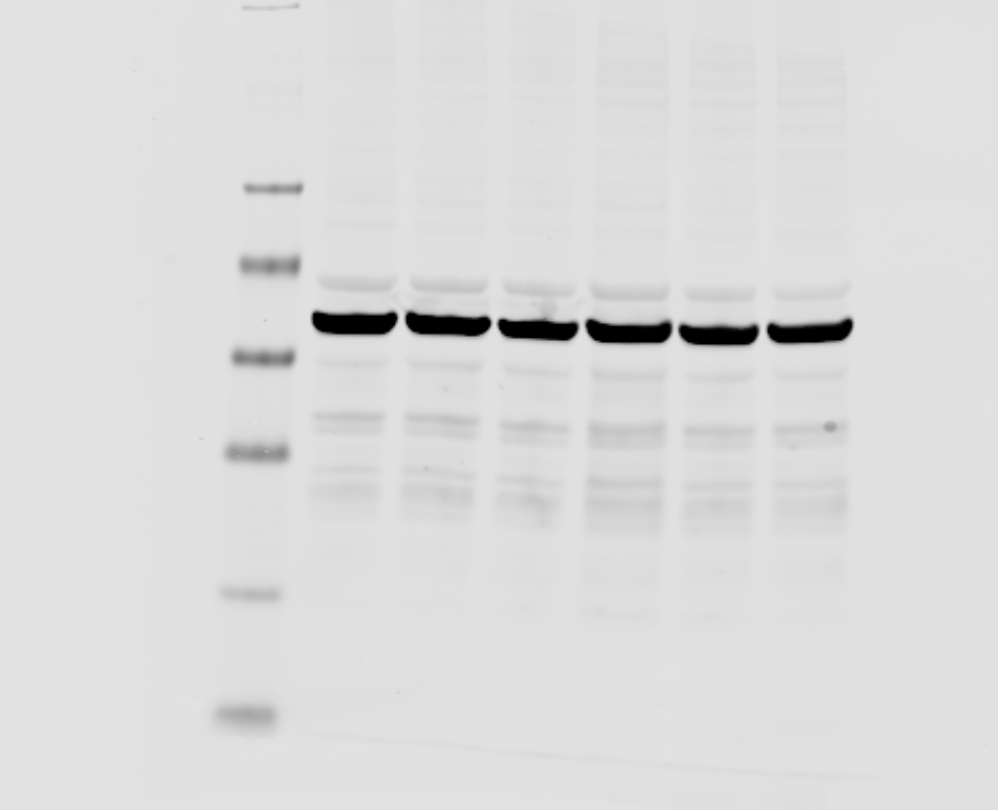

Supplement: Figure 1—source data 1. [file elife-96992-fig1-data1.zip › Figure 1-Figure Supplement 2-Source data 1. Uncropped and labelled gels for Figure 1-Figure Supplement 2/Figure 1-figure supplement 2 blots.docx]
