# Supplementary figures and images for: Inhibition of ULK1/2 and KRASG12C controls tumor growth in preclinical models of lung cancer
Source: eLife. 2024 Aug 30;13:RP96992. doi: 10.7554/eLife.96992 (PMC11364435; doi:10.7554/eLife.96992)

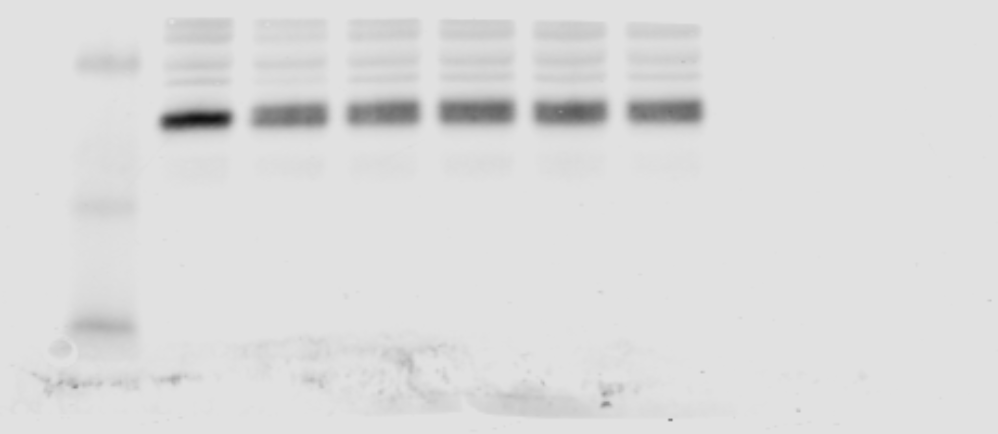

Supplement: Figure 1—source data 2. [file elife-96992-fig1-data2.zip › Figure 1-Figure Supplement 2-Source data 2. Raw unedited gels for Figure 1-Figure supplement 2/panel H/RAS.tif]

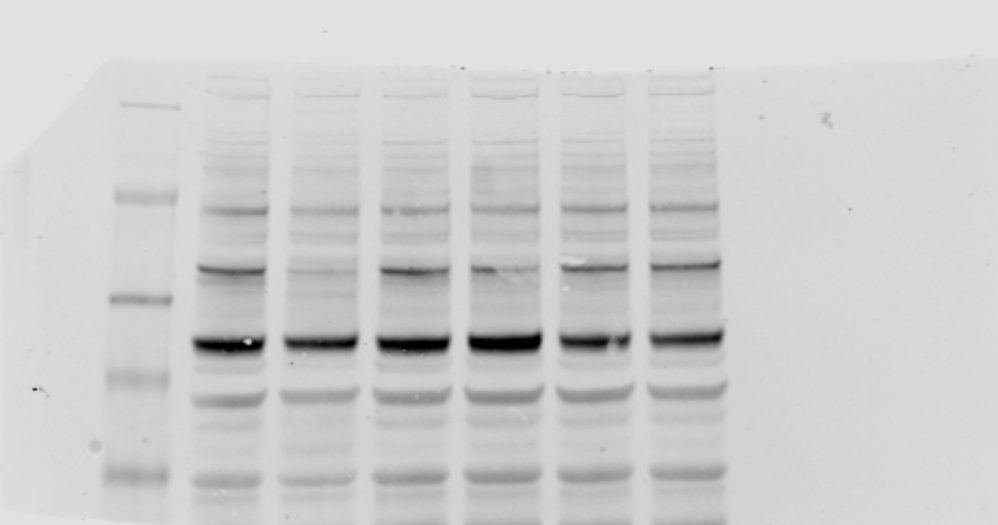

Supplement: Figure 1—source data 2. [file elife-96992-fig1-data2.zip › Figure 1-Figure Supplement 2-Source data 2. Raw unedited gels for Figure 1-Figure supplement 2/panel H/p-AKT.tif]

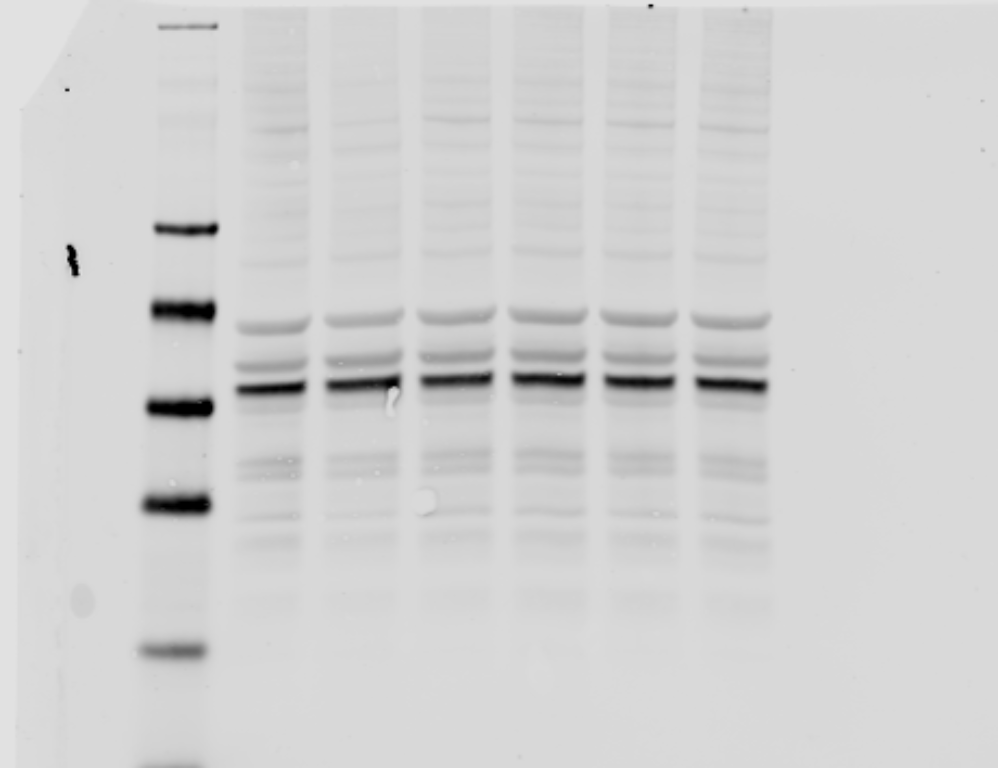

Supplement: Figure 1—source data 2. [file elife-96992-fig1-data2.zip › Figure 1-Figure Supplement 2-Source data 2. Raw unedited gels for Figure 1-Figure supplement 2/panel H/t-ERK.tif]

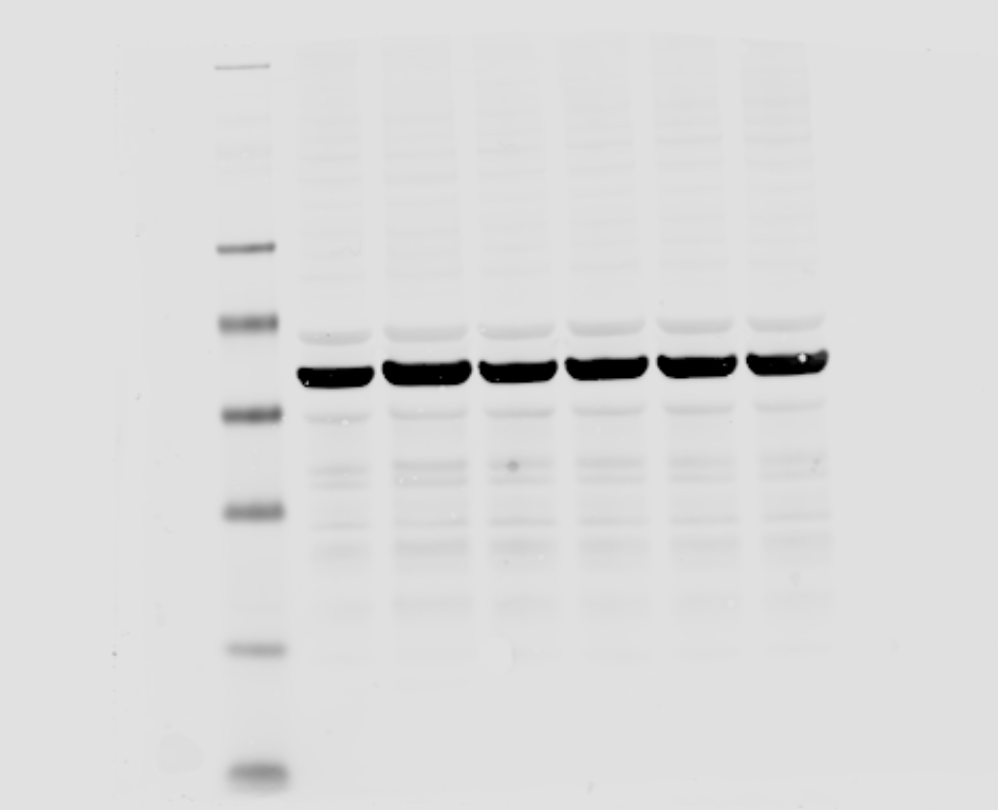

Supplement: Figure 1—source data 2. [file elife-96992-fig1-data2.zip › Figure 1-Figure Supplement 2-Source data 2. Raw unedited gels for Figure 1-Figure supplement 2/panel H/b-actin.tif]

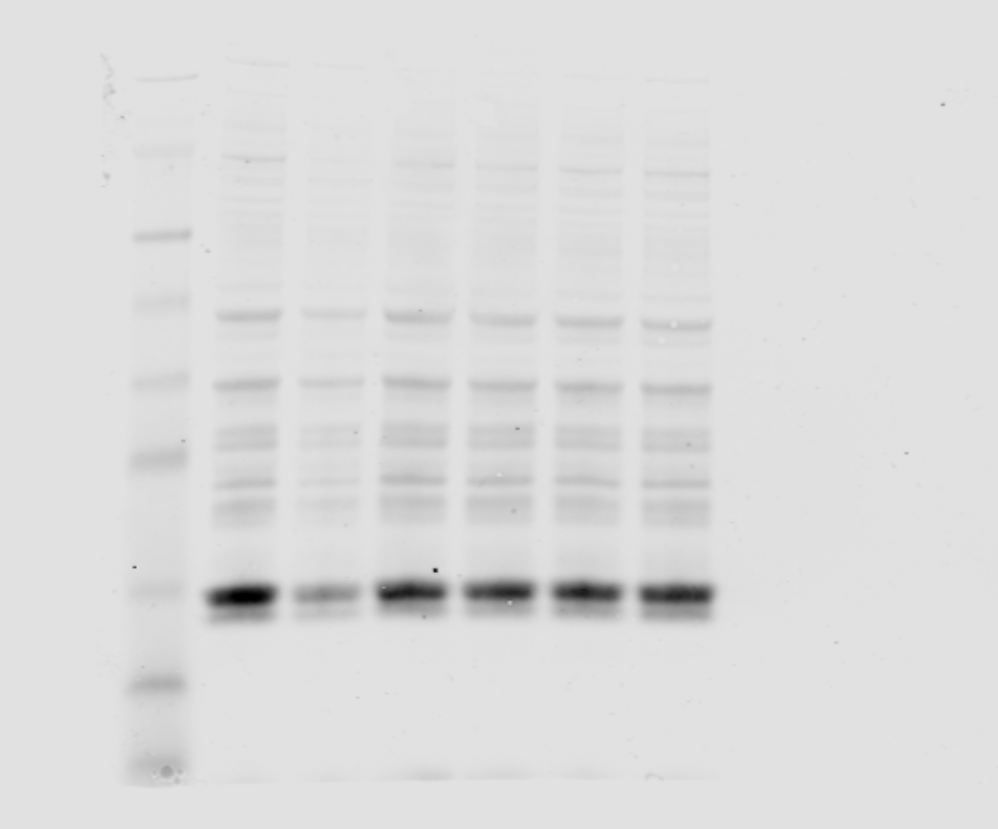

Supplement: Figure 1—source data 2. [file elife-96992-fig1-data2.zip › Figure 1-Figure Supplement 2-Source data 2. Raw unedited gels for Figure 1-Figure supplement 2/panel H/LC3.tif]

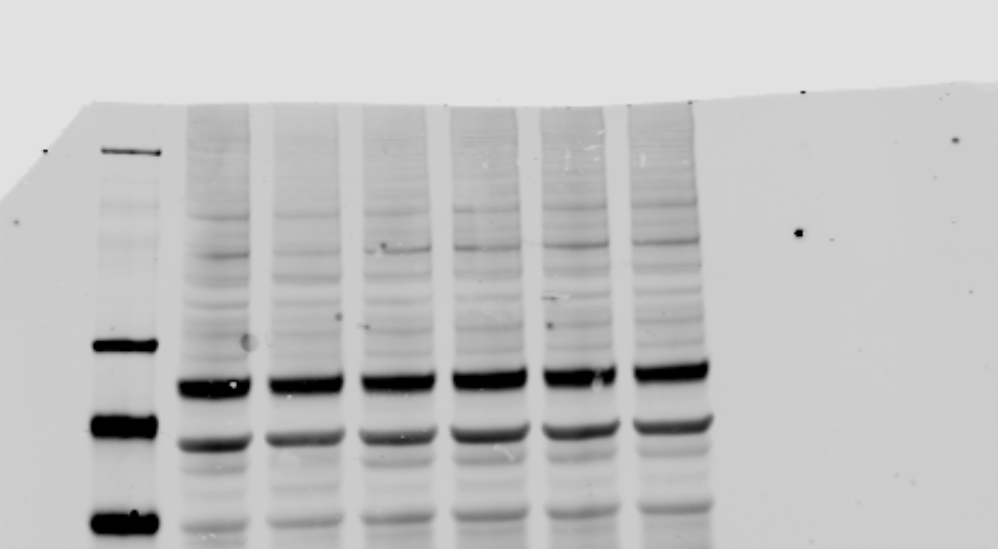

Supplement: Figure 1—source data 2. [file elife-96992-fig1-data2.zip › Figure 1-Figure Supplement 2-Source data 2. Raw unedited gels for Figure 1-Figure supplement 2/panel H/t-AKT.tif]

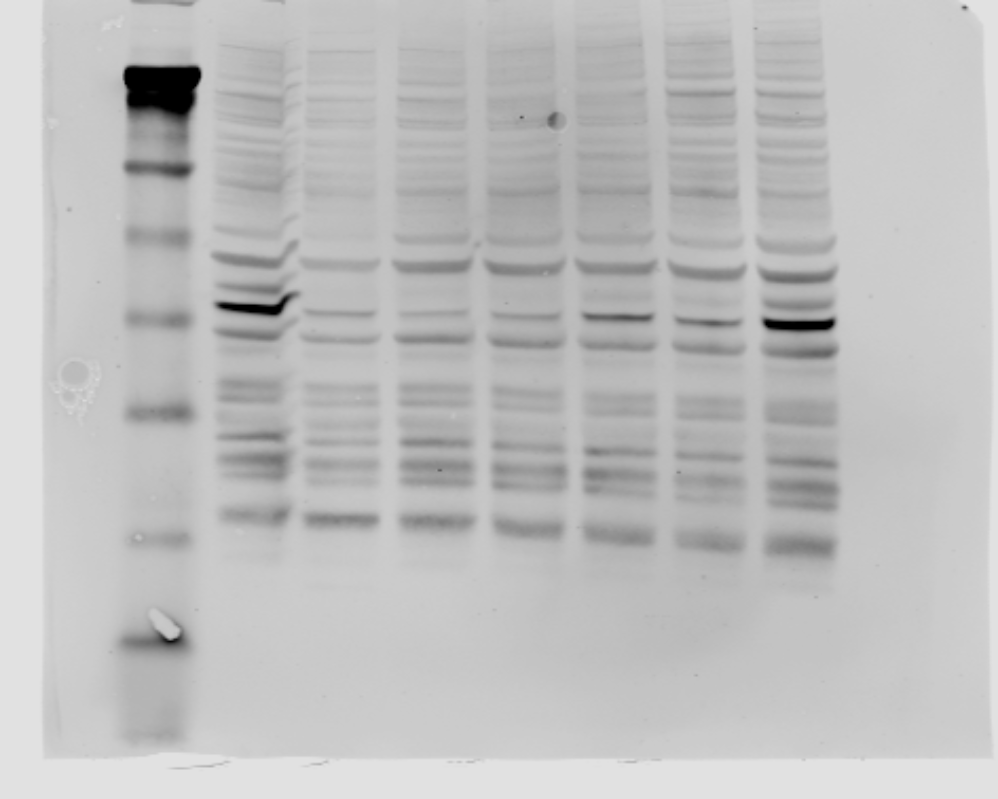

Supplement: Figure 1—source data 2. [file elife-96992-fig1-data2.zip › Figure 1-Figure Supplement 2-Source data 2. Raw unedited gels for Figure 1-Figure supplement 2/panel H/p-ERK.tif]

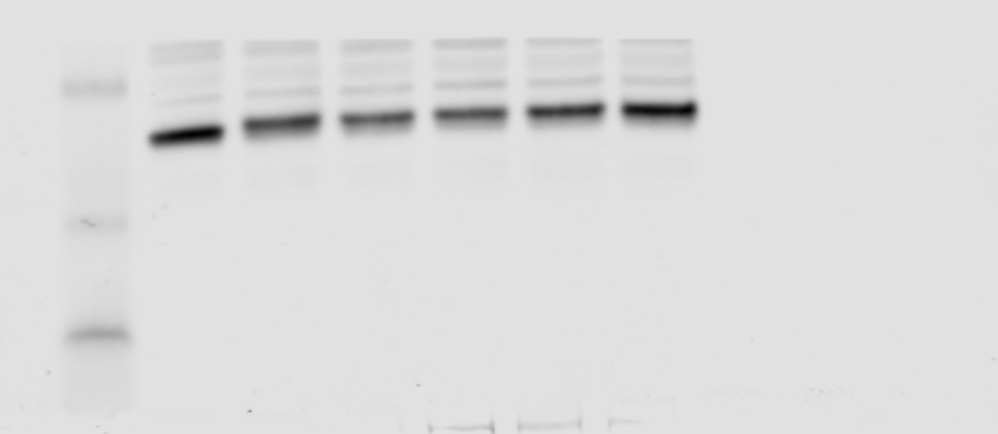

Supplement: Figure 1—source data 2. [file elife-96992-fig1-data2.zip › Figure 1-Figure Supplement 2-Source data 2. Raw unedited gels for Figure 1-Figure supplement 2/panel I/RAS.tif]

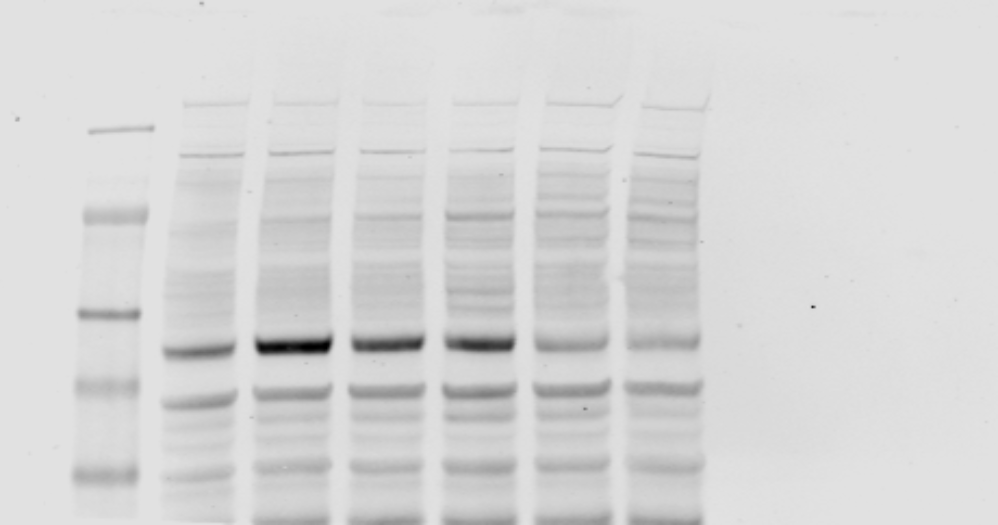

Supplement: Figure 1—source data 2. [file elife-96992-fig1-data2.zip › Figure 1-Figure Supplement 2-Source data 2. Raw unedited gels for Figure 1-Figure supplement 2/panel I/p-AKT.tif]

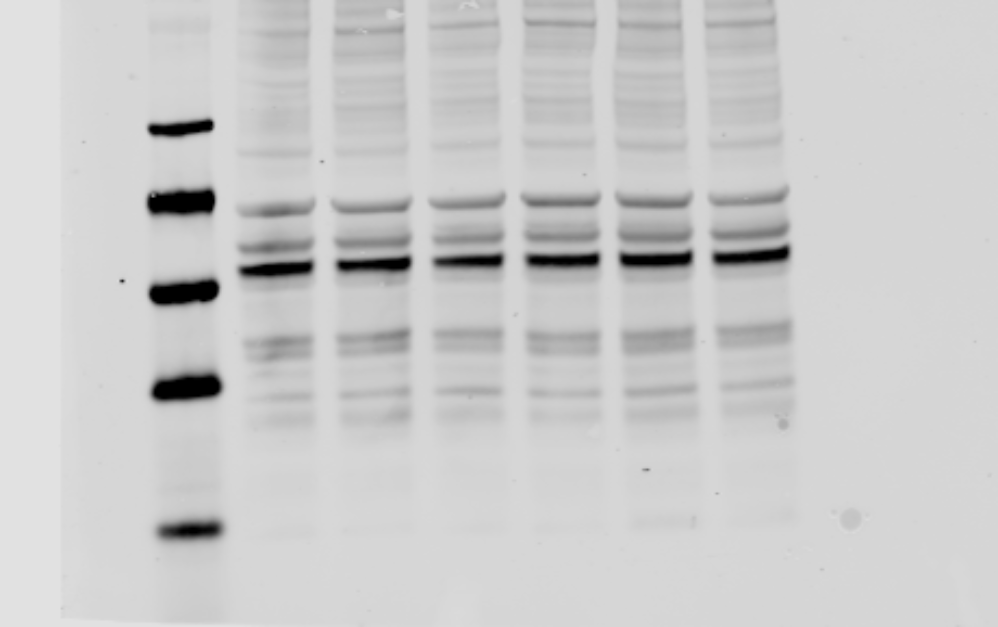

Supplement: Figure 1—source data 2. [file elife-96992-fig1-data2.zip › Figure 1-Figure Supplement 2-Source data 2. Raw unedited gels for Figure 1-Figure supplement 2/panel I/t-ERK.tif]

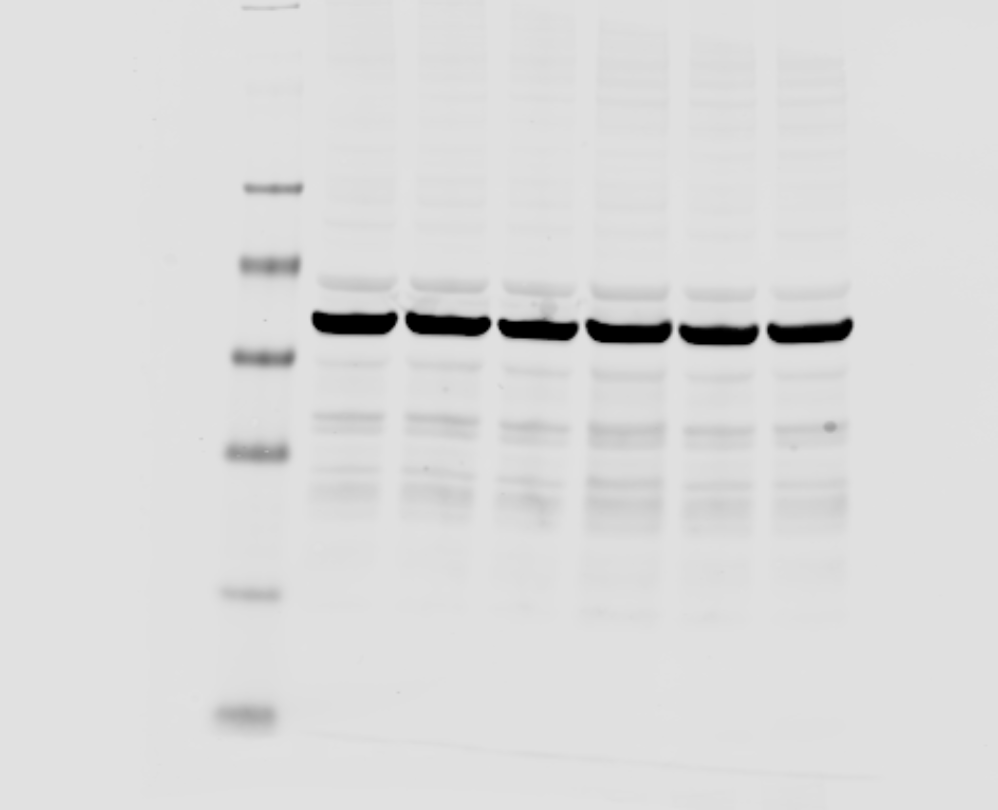

Supplement: Figure 1—source data 2. [file elife-96992-fig1-data2.zip › Figure 1-Figure Supplement 2-Source data 2. Raw unedited gels for Figure 1-Figure supplement 2/panel I/b-actin.tif]

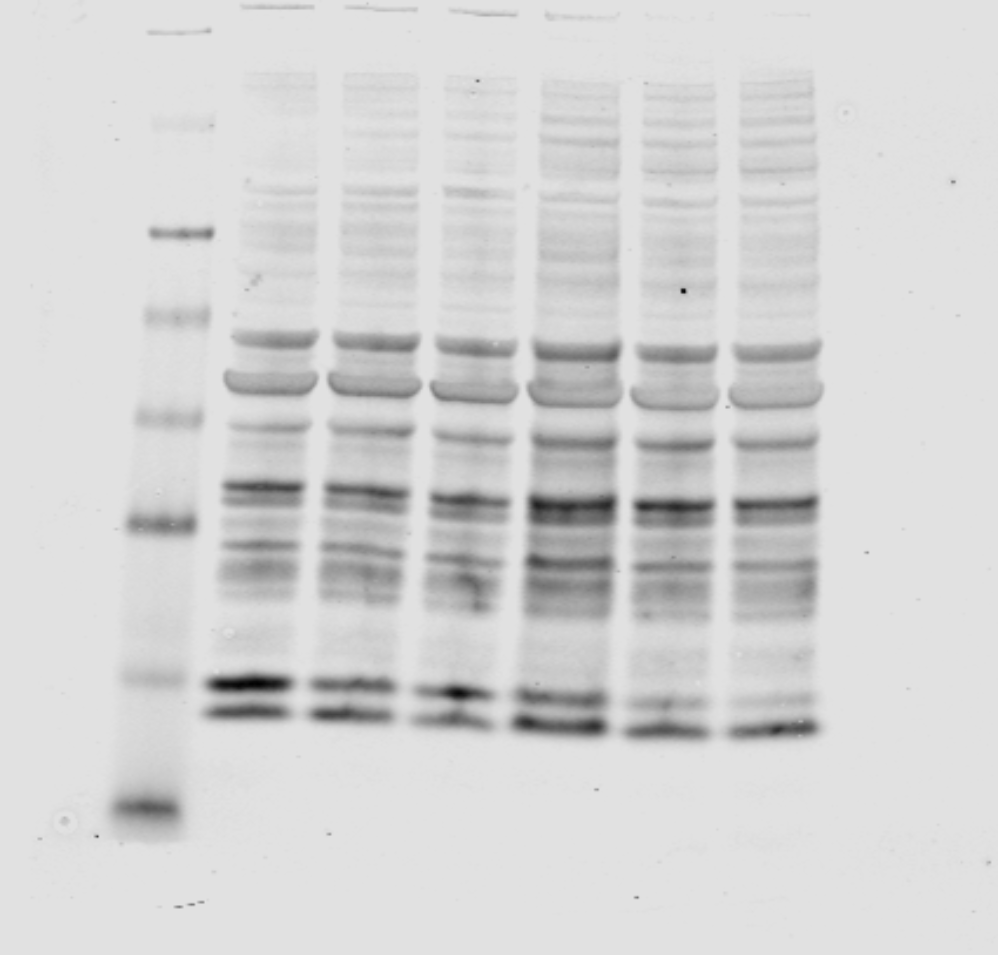

Supplement: Figure 1—source data 2. [file elife-96992-fig1-data2.zip › Figure 1-Figure Supplement 2-Source data 2. Raw unedited gels for Figure 1-Figure supplement 2/panel I/LC3.tif]

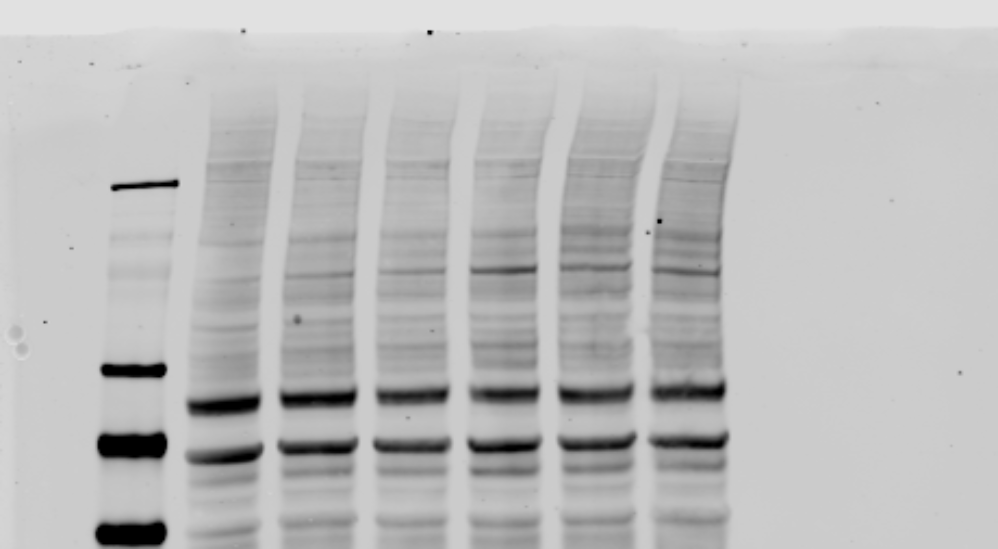

Supplement: Figure 1—source data 2. [file elife-96992-fig1-data2.zip › Figure 1-Figure Supplement 2-Source data 2. Raw unedited gels for Figure 1-Figure supplement 2/panel I/t-AKT.tif]

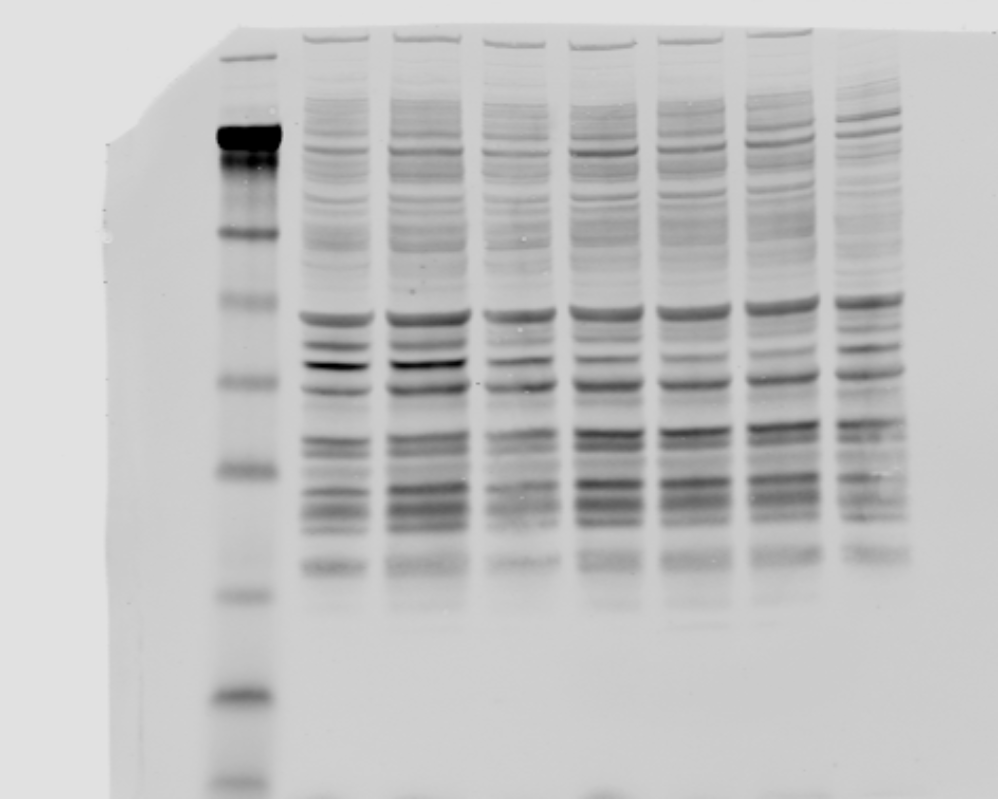

Supplement: Figure 1—source data 2. [file elife-96992-fig1-data2.zip › Figure 1-Figure Supplement 2-Source data 2. Raw unedited gels for Figure 1-Figure supplement 2/panel I/p-ERK.tif]

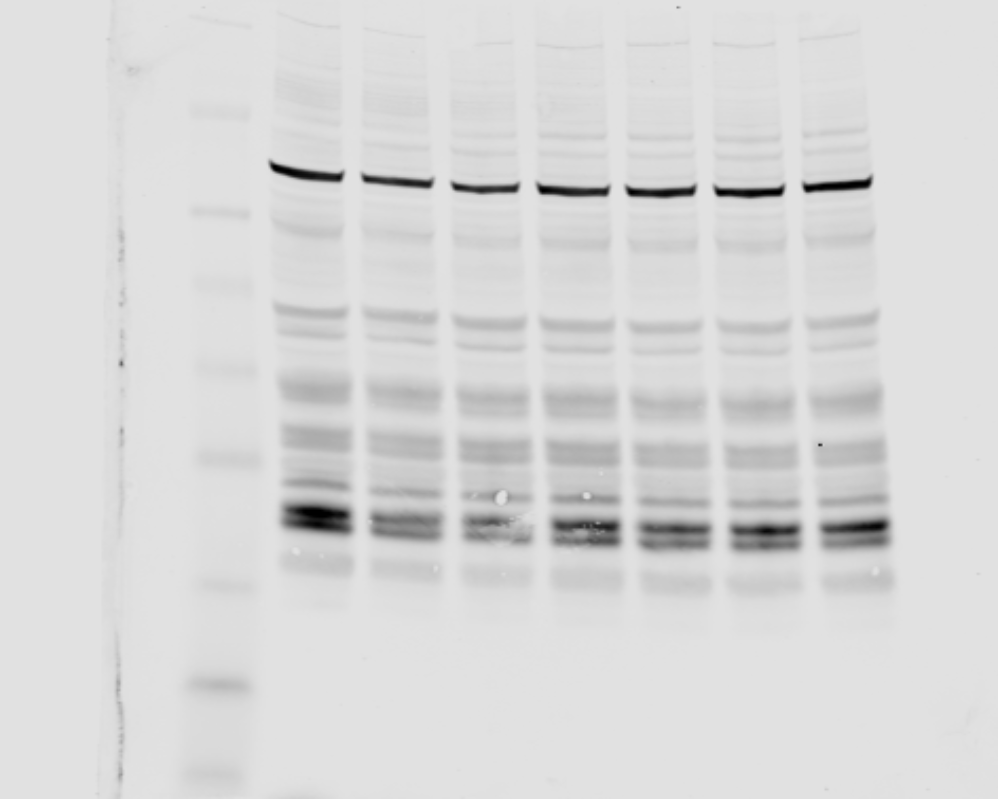

Supplement: Figure 1—source data 2. [file elife-96992-fig1-data2.zip › Figure 1-Figure Supplement 2-Source data 2. Raw unedited gels for Figure 1-Figure supplement 2/panel G/RAS.tif]

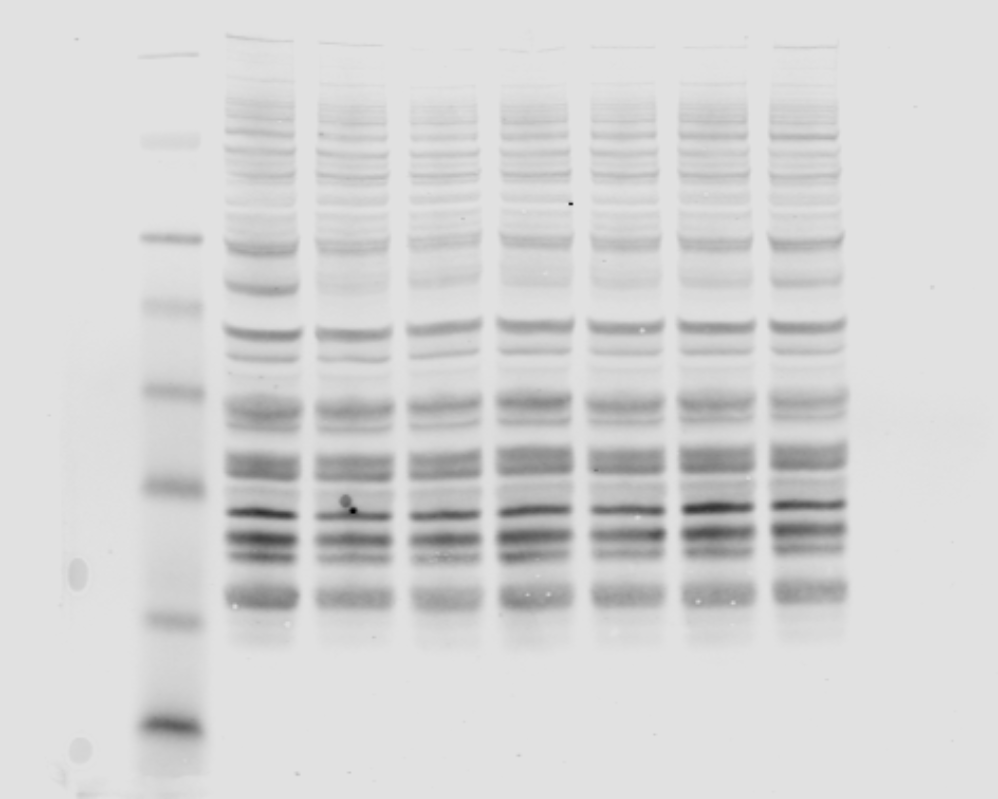

Supplement: Figure 1—source data 2. [file elife-96992-fig1-data2.zip › Figure 1-Figure Supplement 2-Source data 2. Raw unedited gels for Figure 1-Figure supplement 2/panel G/p-AKT.tif]

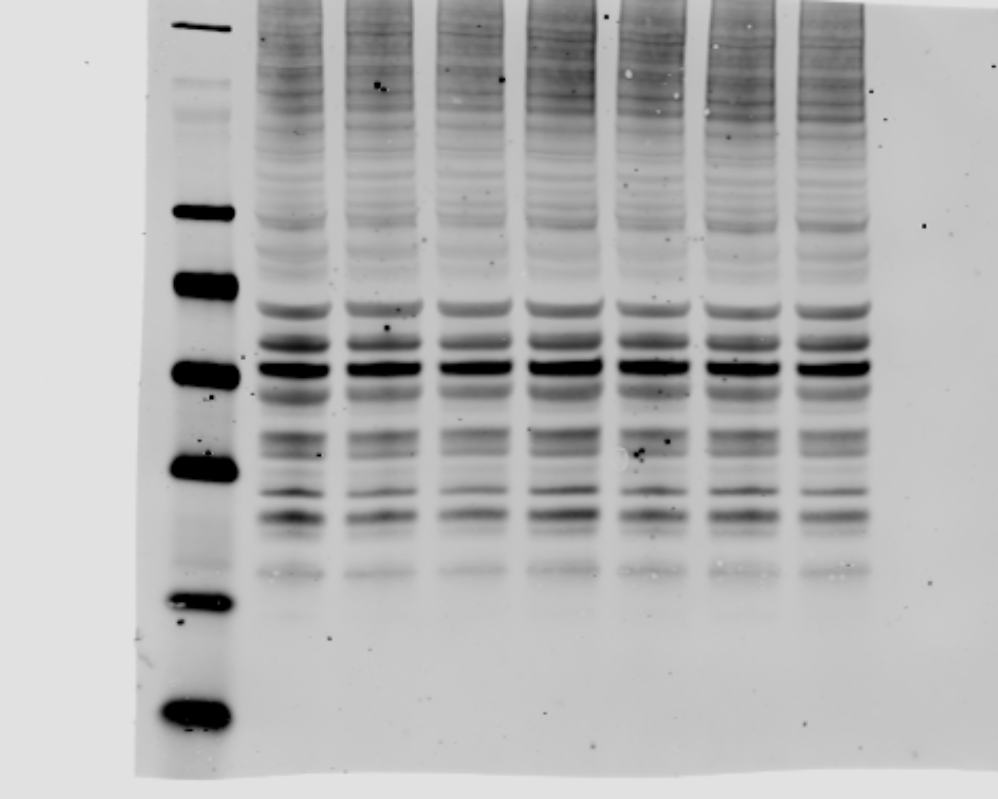

Supplement: Figure 1—source data 2. [file elife-96992-fig1-data2.zip › Figure 1-Figure Supplement 2-Source data 2. Raw unedited gels for Figure 1-Figure supplement 2/panel G/t-ERK.tif]

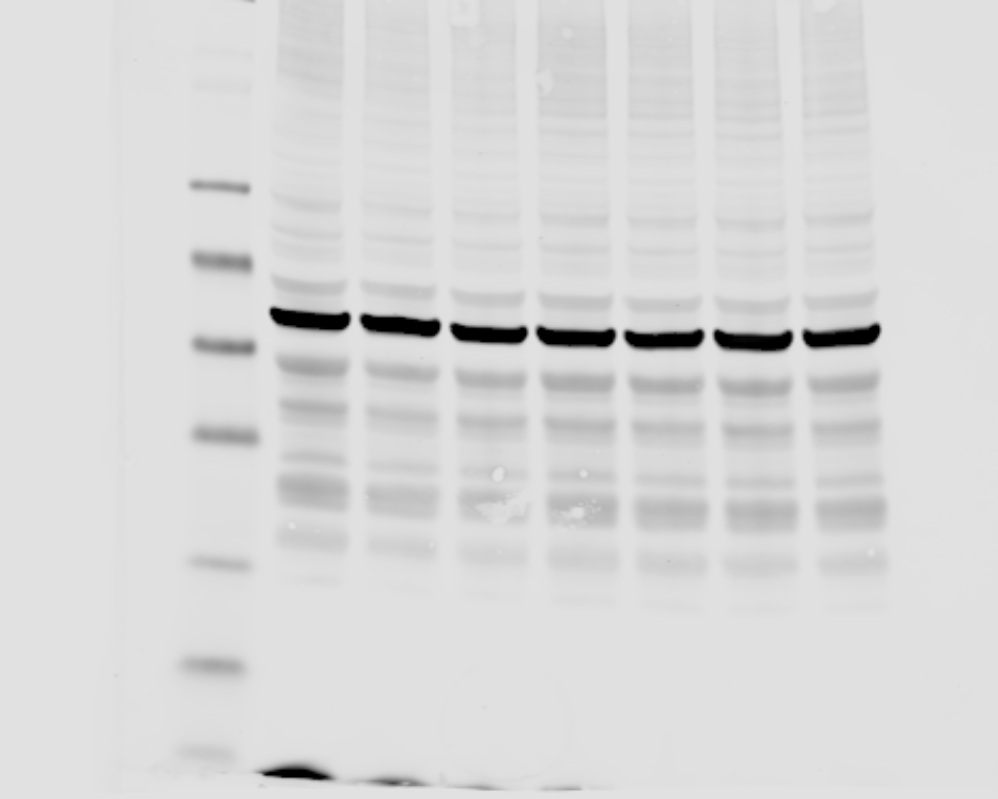

Supplement: Figure 1—source data 2. [file elife-96992-fig1-data2.zip › Figure 1-Figure Supplement 2-Source data 2. Raw unedited gels for Figure 1-Figure supplement 2/panel G/b-actin.tif]

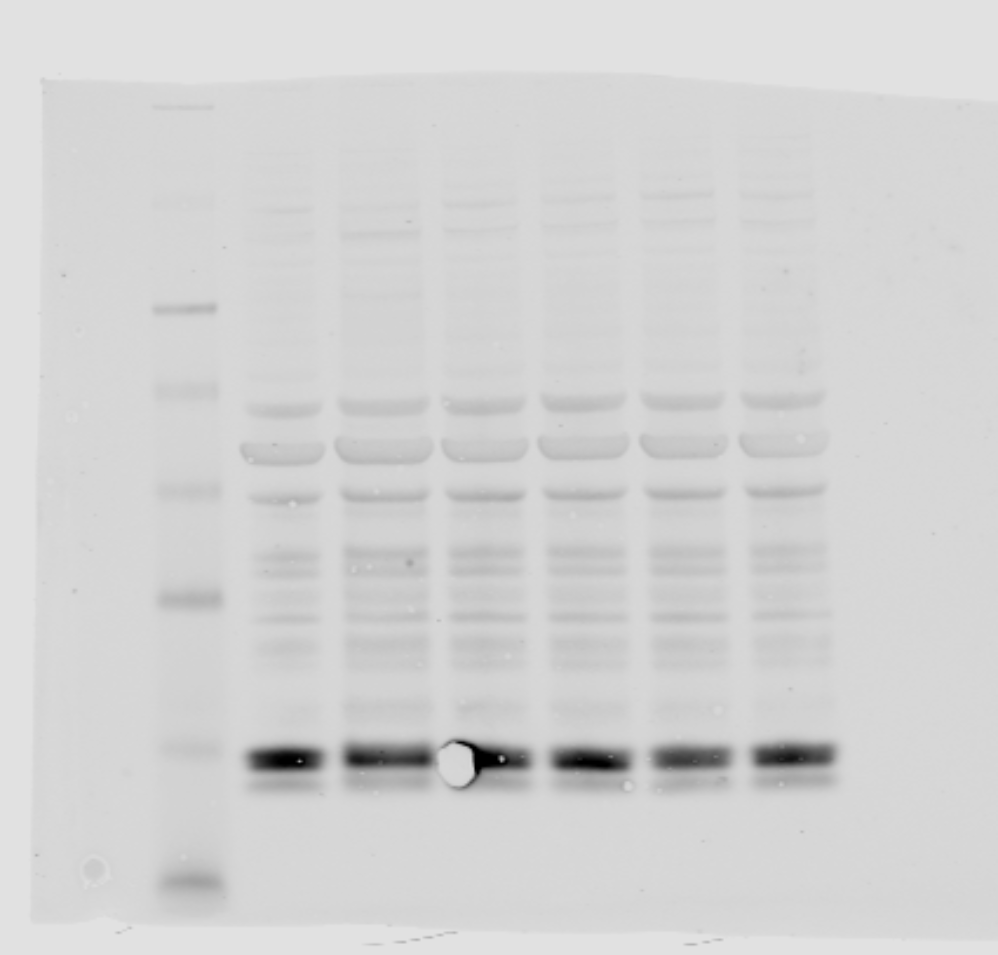

Supplement: Figure 1—source data 2. [file elife-96992-fig1-data2.zip › Figure 1-Figure Supplement 2-Source data 2. Raw unedited gels for Figure 1-Figure supplement 2/panel G/LC3AB.tif]

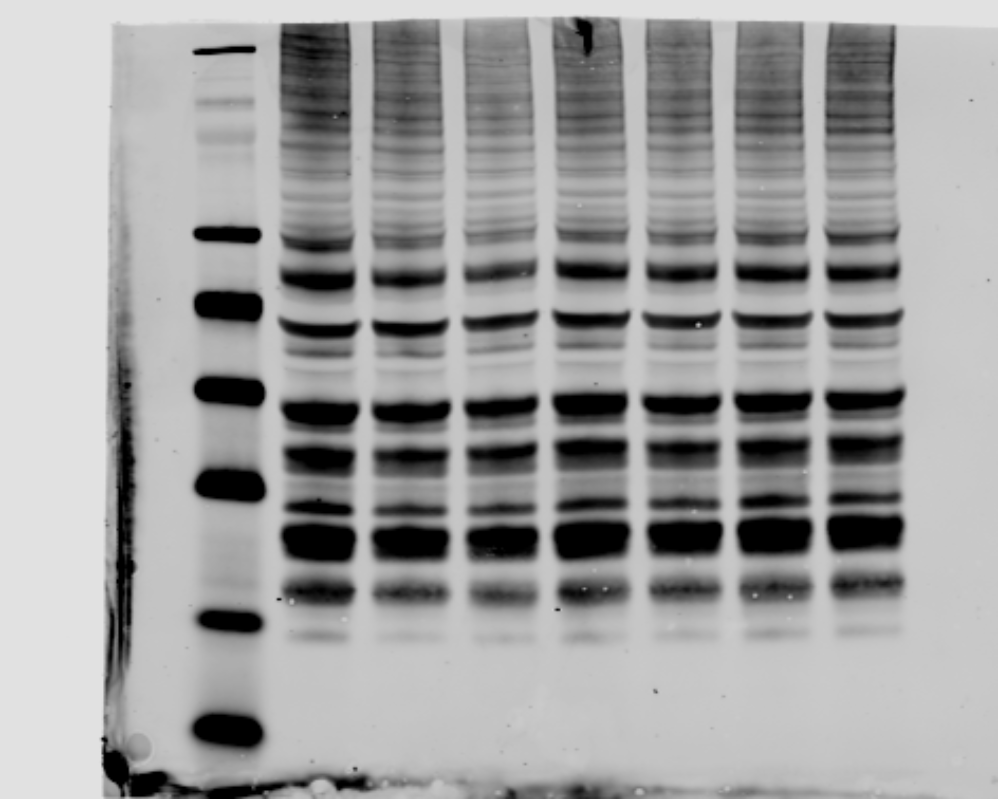

Supplement: Figure 1—source data 2. [file elife-96992-fig1-data2.zip › Figure 1-Figure Supplement 2-Source data 2. Raw unedited gels for Figure 1-Figure supplement 2/panel G/t-AKT.tif]

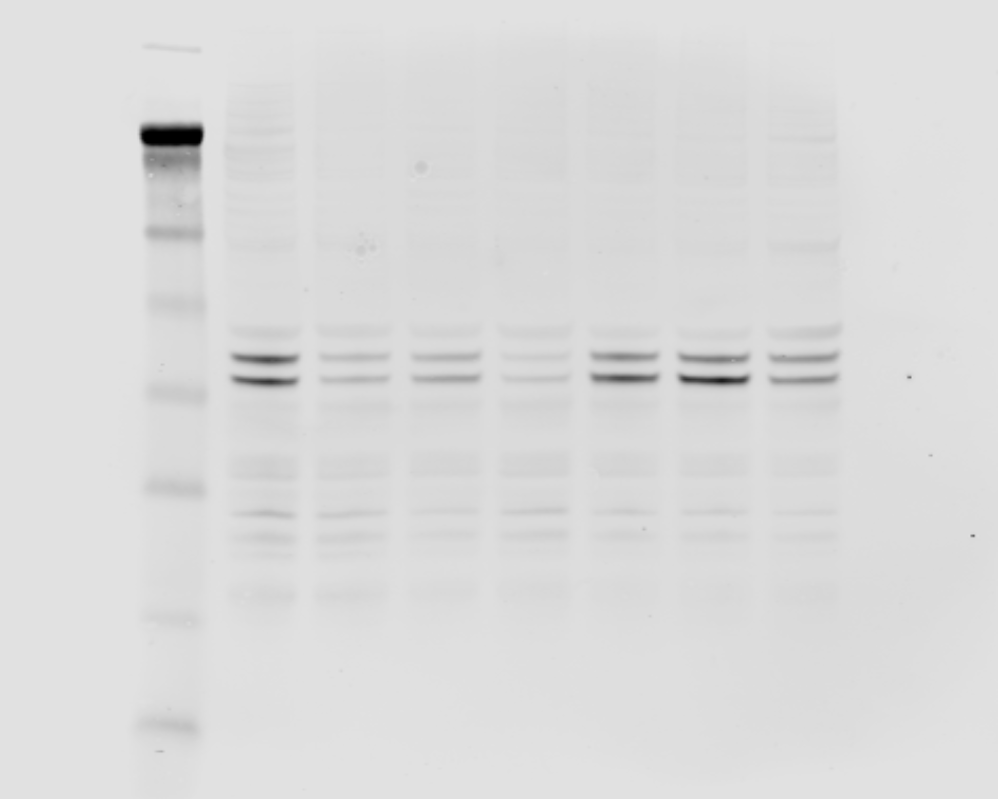

Supplement: Figure 1—source data 2. [file elife-96992-fig1-data2.zip › Figure 1-Figure Supplement 2-Source data 2. Raw unedited gels for Figure 1-Figure supplement 2/panel G/p-ERK.tif]

**Figure 2 A:**

pULK1 S555


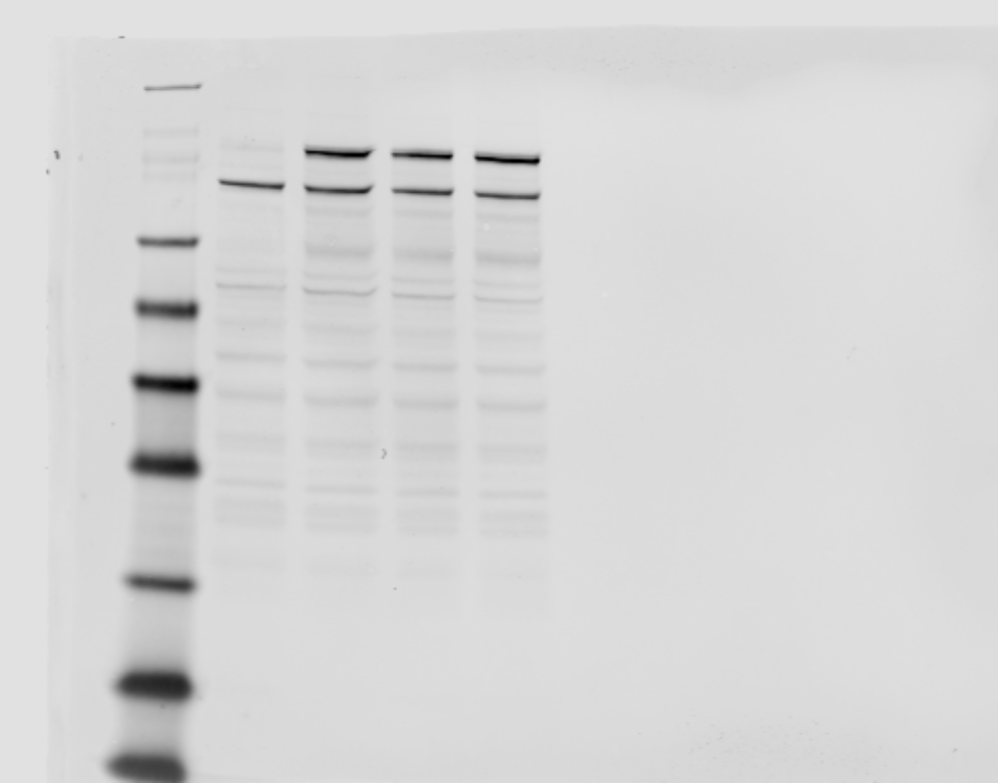


PULK1 S757


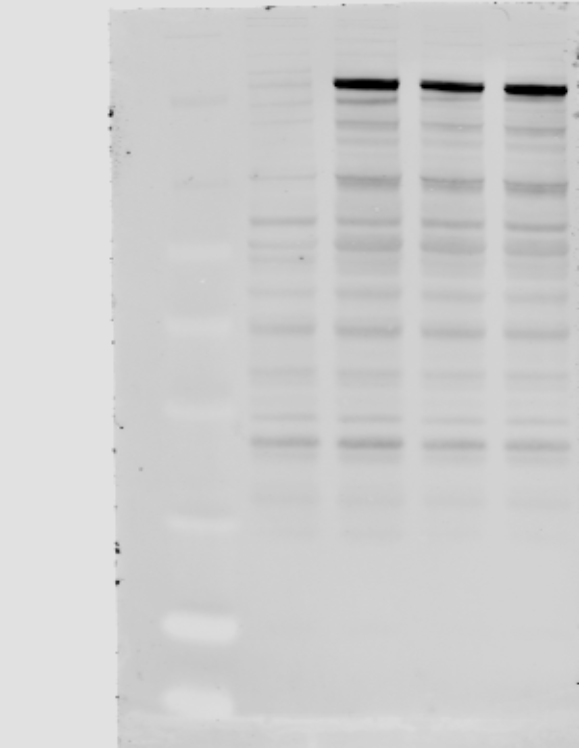


ULK1


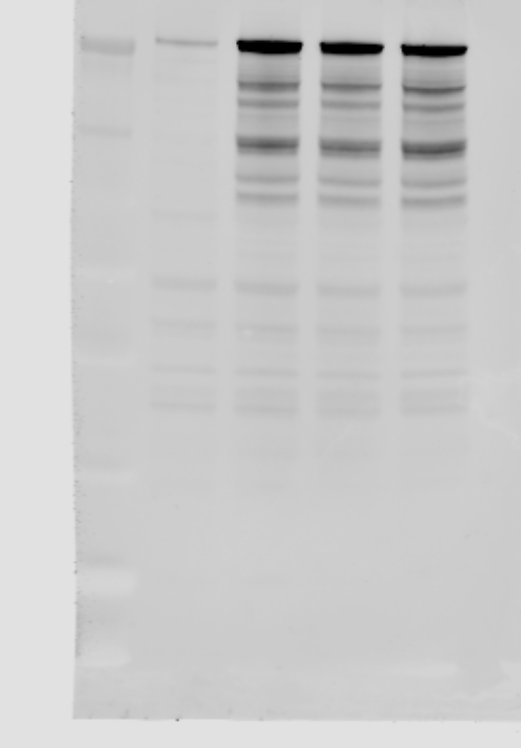


HA


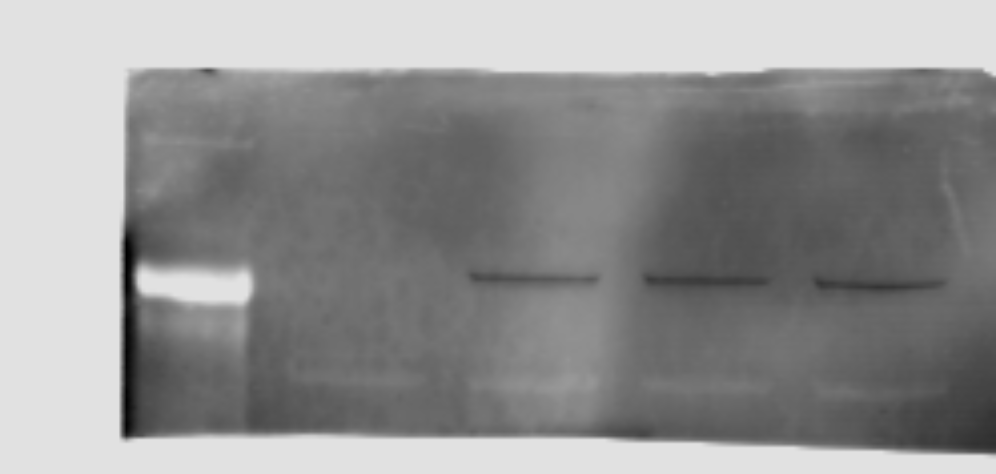


B-actin


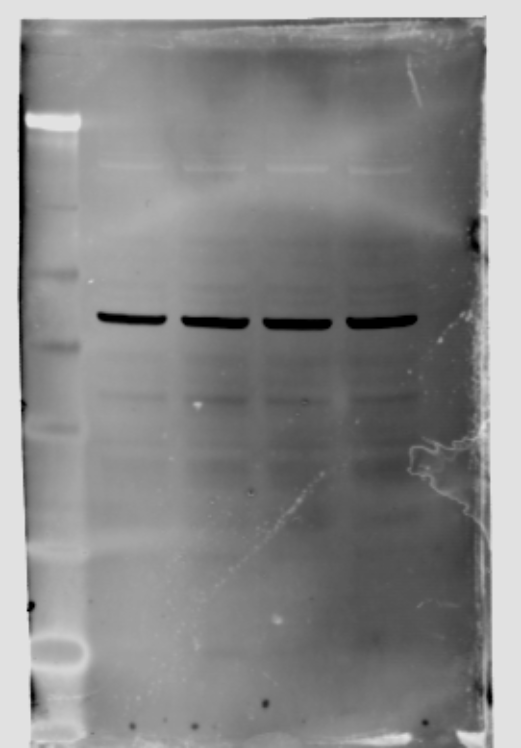


**Figure 2 B**

pULK1 S555


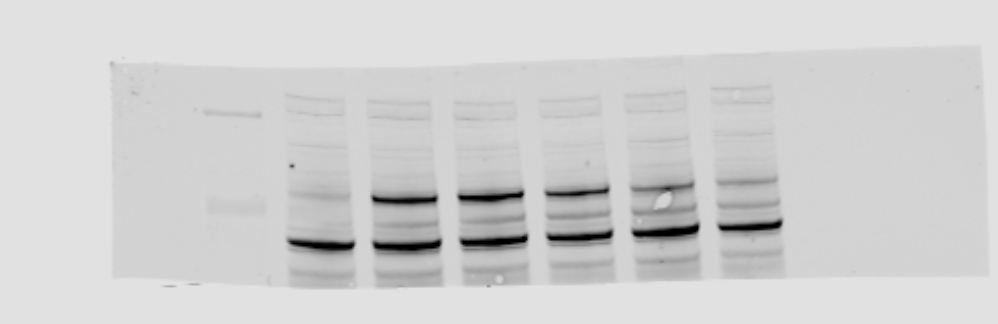


pULK1 S757


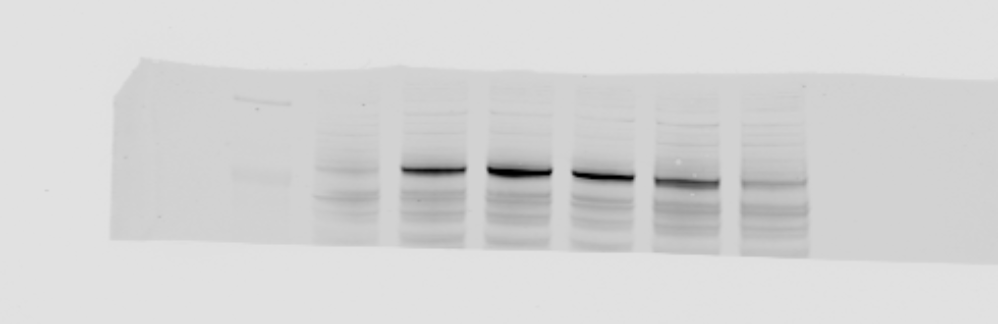


ULK1


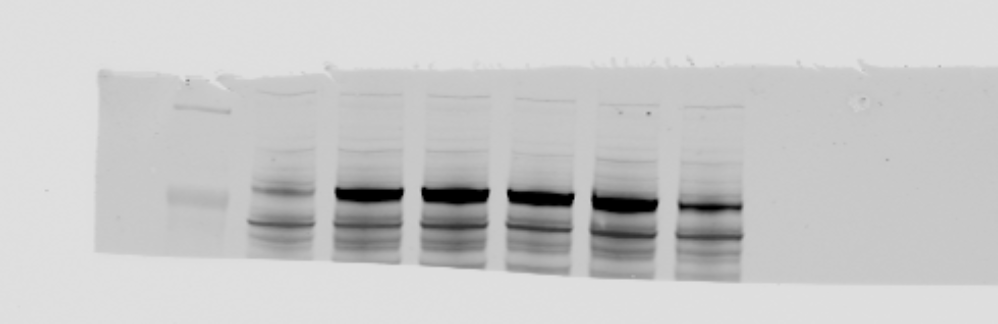


HA


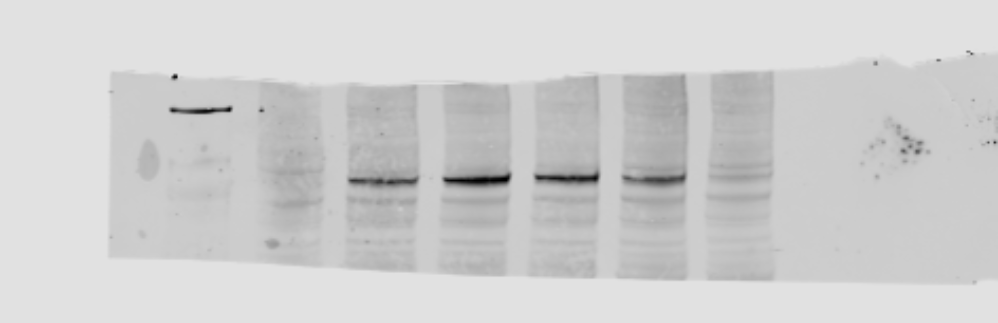


B-actin


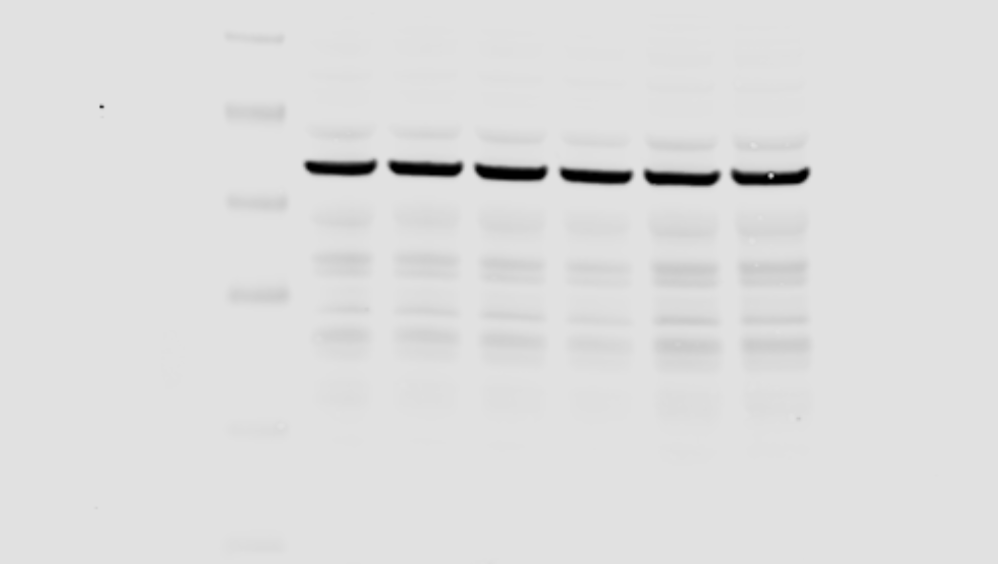

Supplement: Figure 2—source data 1. [file elife-96992-fig2-data1.zip › Figure 2-Source data 1. Uncropped and labelled gels for Figure 2/Figure 2 blots.docx]

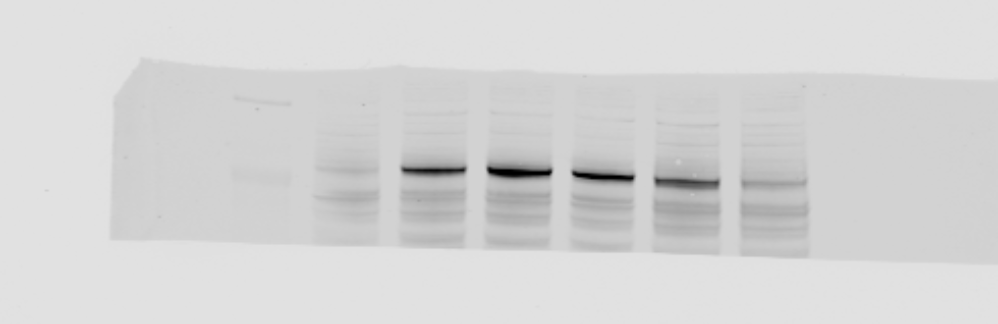

Supplement: Figure 2—source data 2. [file elife-96992-fig2-data2.zip › Figure 2-Source data 2. Raw unedited gels for Figure 2/panel B/pULK1 S757.tif]

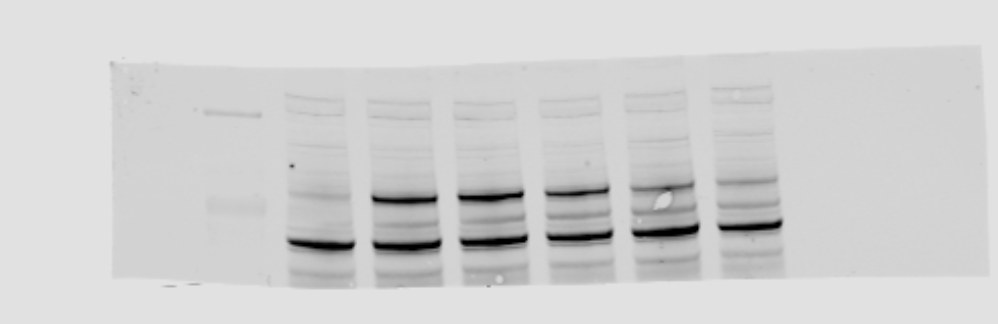

Supplement: Figure 2—source data 2. [file elife-96992-fig2-data2.zip › Figure 2-Source data 2. Raw unedited gels for Figure 2/panel B/pULK1 S555.tif]

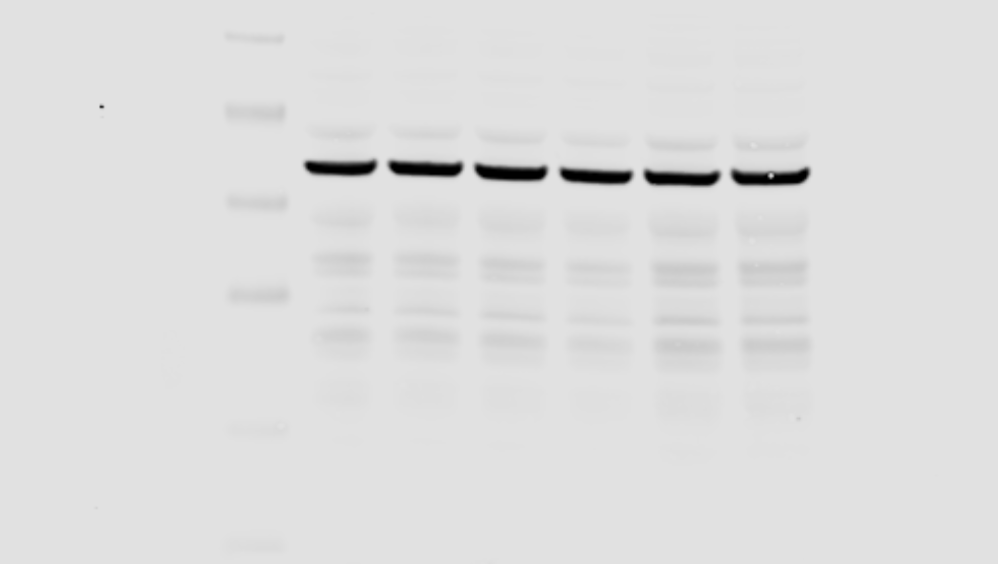

Supplement: Figure 2—source data 2. [file elife-96992-fig2-data2.zip › Figure 2-Source data 2. Raw unedited gels for Figure 2/panel B/b-actin.tif]

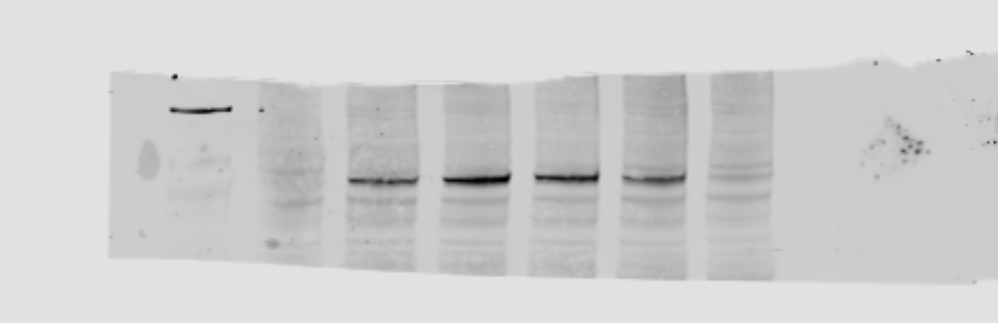

Supplement: Figure 2—source data 2. [file elife-96992-fig2-data2.zip › Figure 2-Source data 2. Raw unedited gels for Figure 2/panel B/HA.tif]

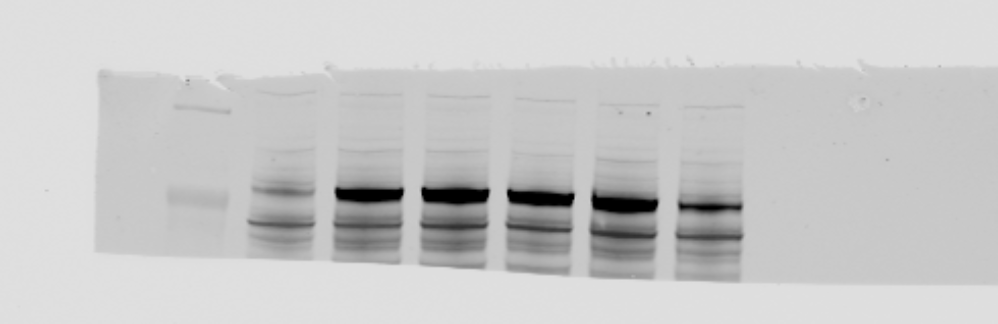

Supplement: Figure 2—source data 2. [file elife-96992-fig2-data2.zip › Figure 2-Source data 2. Raw unedited gels for Figure 2/panel B/ULK1.tif]

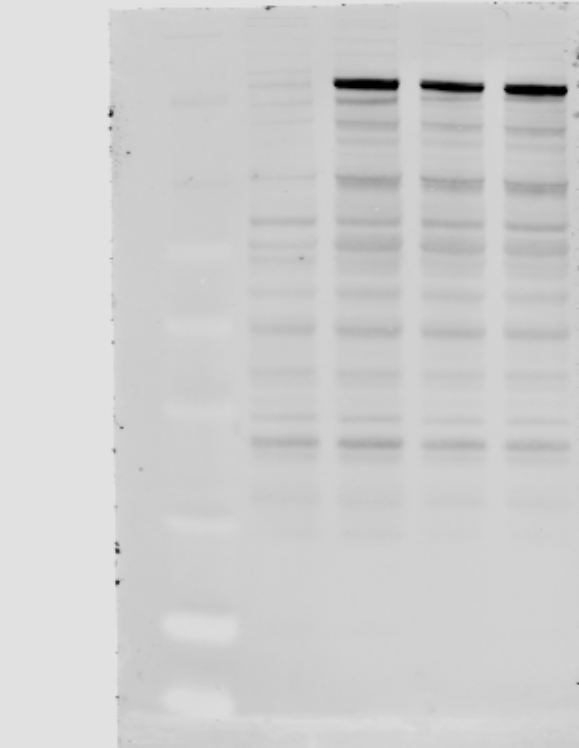

Supplement: Figure 2—source data 2. [file elife-96992-fig2-data2.zip › Figure 2-Source data 2. Raw unedited gels for Figure 2/panel A/pULK1 S757.tif]

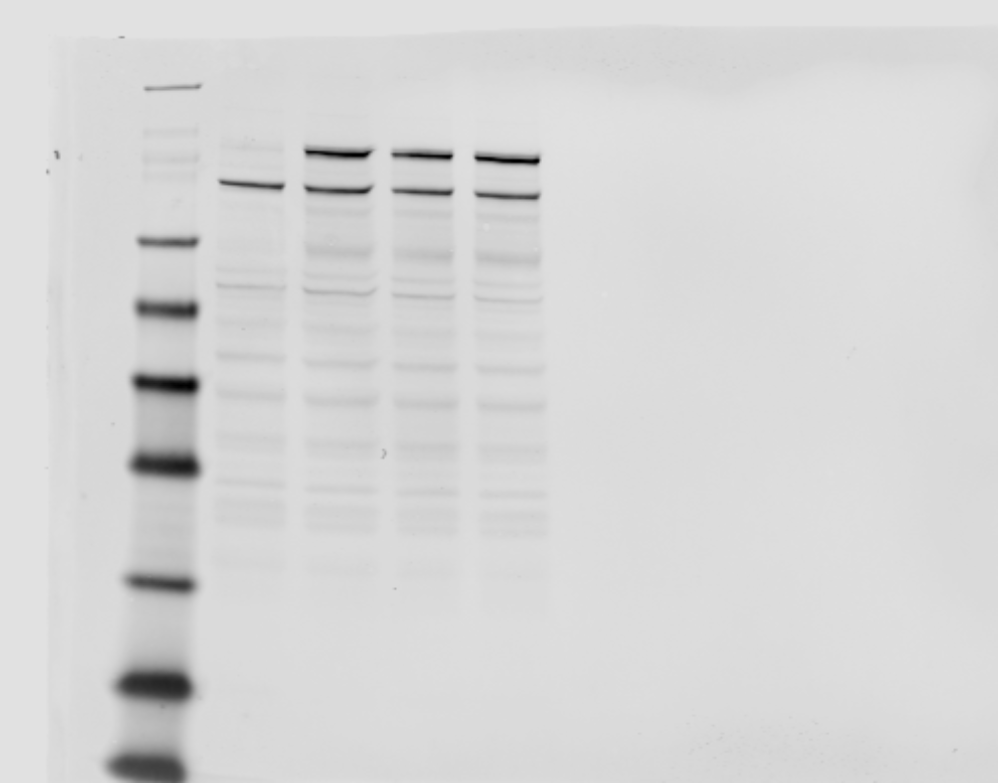

Supplement: Figure 2—source data 2. [file elife-96992-fig2-data2.zip › Figure 2-Source data 2. Raw unedited gels for Figure 2/panel A/pULK1 s555.tif]

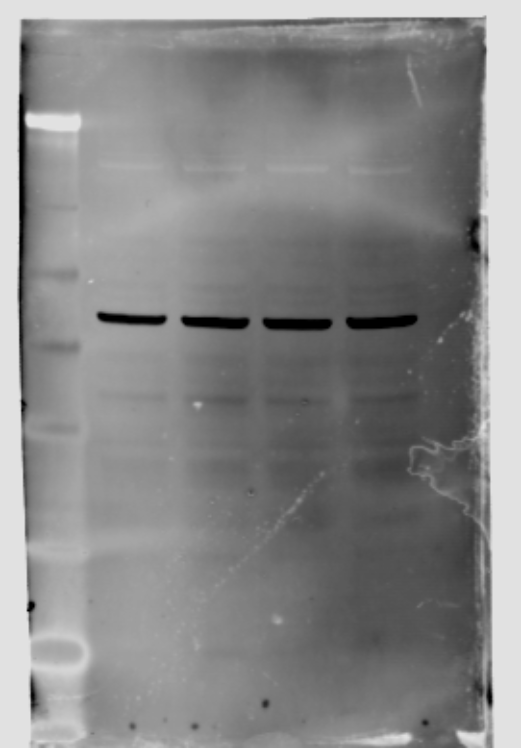

Supplement: Figure 2—source data 2. [file elife-96992-fig2-data2.zip › Figure 2-Source data 2. Raw unedited gels for Figure 2/panel A/b-actin.tif]

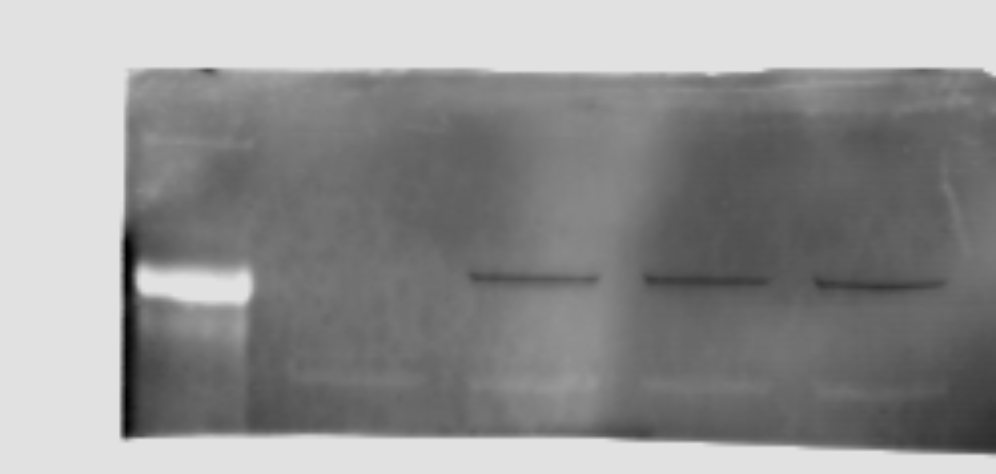

Supplement: Figure 2—source data 2. [file elife-96992-fig2-data2.zip › Figure 2-Source data 2. Raw unedited gels for Figure 2/panel A/HA.tif]

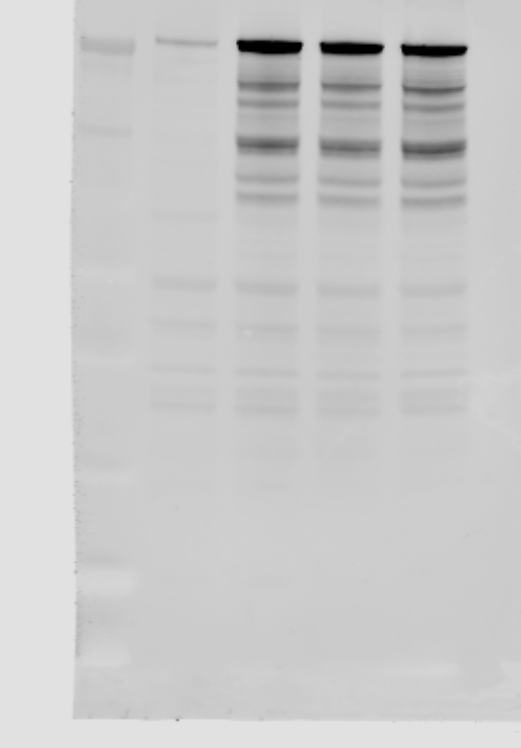

Supplement: Figure 2—source data 2. [file elife-96992-fig2-data2.zip › Figure 2-Source data 2. Raw unedited gels for Figure 2/panel A/ULK1.tif]

**Figure 6: A**

pERK


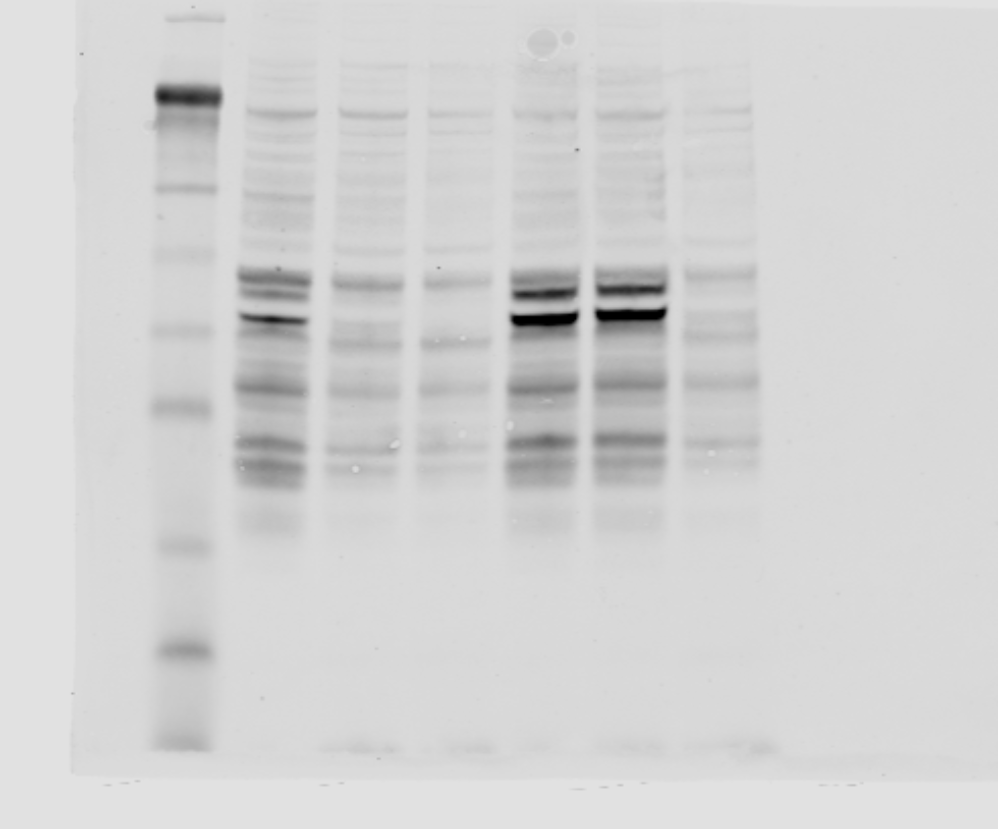


tERK


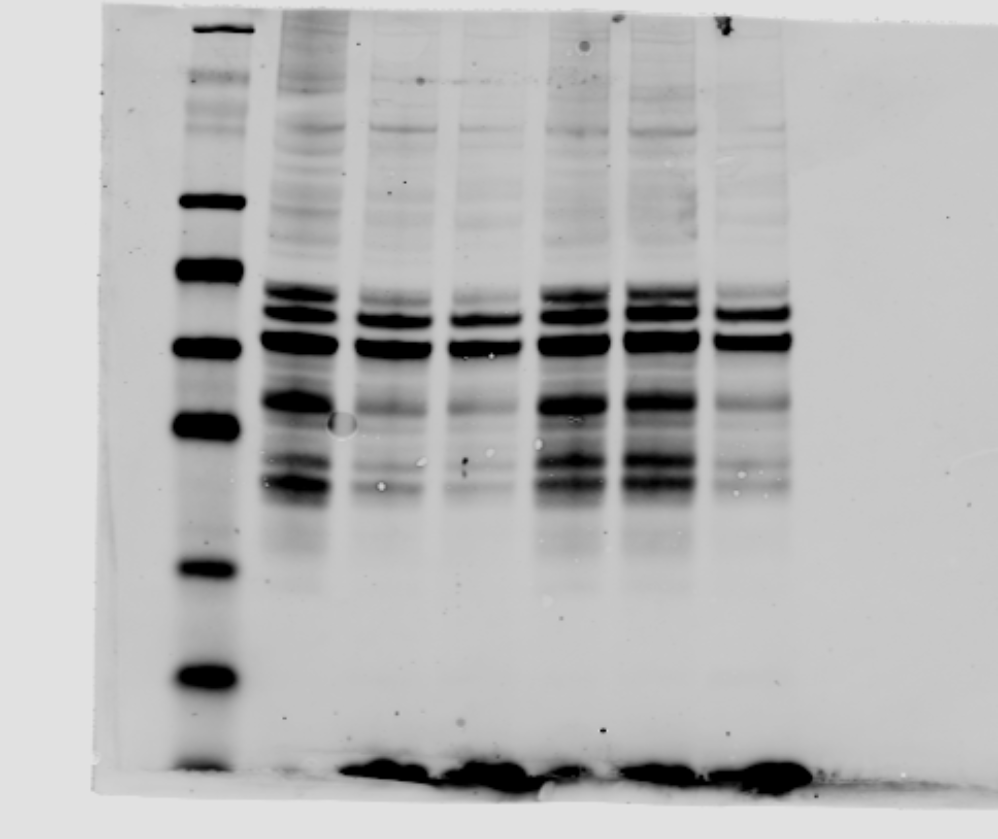


RAS


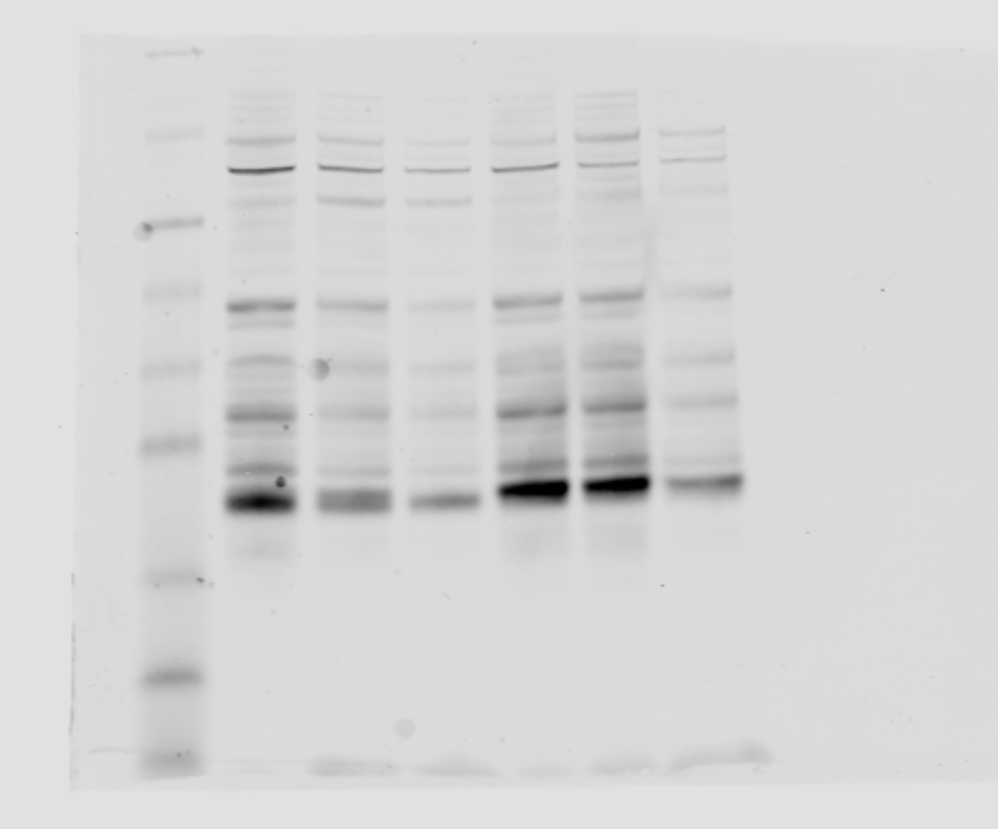


b-actin


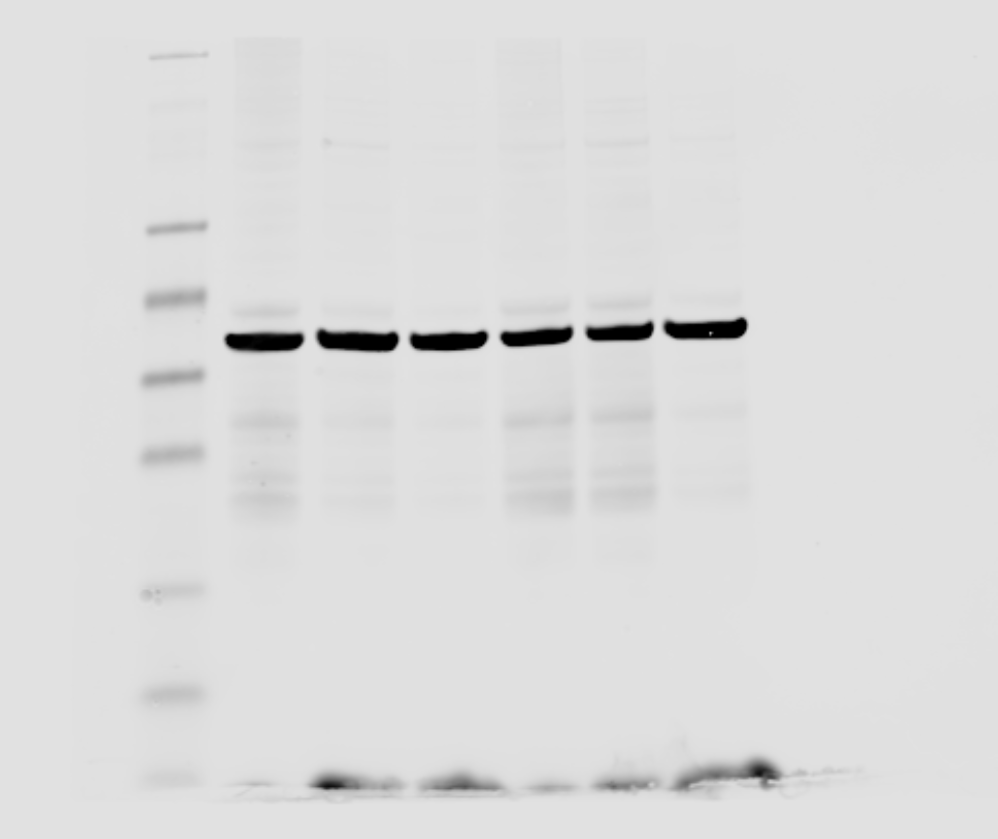

Supplement: Figure 6—source data 1. [file elife-96992-fig6-data1.zip › Figure 6-Source data 1. Uncropped and labelled gels for Figure 6/Figure 6 blots.docx]
